# Supplementary material for: IMAGE: high-powered detection of genetic effects on DNA methylation using integrated methylation QTL mapping and allele-specific analysis
Source: Genome Biol. 2019 Oct 24;20:220. doi: 10.1186/s13059-019-1813-1 (PMC6813132; doi:10.1186/s13059-019-1813-1)
Supplement: Supplementary file 1 — Additional file 1: Supplementary text on IMAGE modeling and inference details. [file 13059_2019_1813_MOESM1_ESM.docx]

**Supplementary Text**

**1. Likelihood Approximation for Homozygous Individuals**

Here, we provide details on the approximation used for homozygous individuals. All notations follow the main text. Recall that we denote $y_{i}$ and $r_{i}$ as the methylated read count and total read count for *i*th individual, for $i=1,\cdots,n$. We denote the corresponding read counts mapped to the haplotype of the two alleles of *i*th individual as $y_{il}$ and $r_{il}$, for $l=1$ or 2; thus we have $y_{i}=y_{i1}+y_{i2}$ and $r_{i}=r_{i1}+r_{i2}$. Recall that, for homozygous individuals, we derive a model on $y_{i}$ and $r_{i}$ by summing over all possible values of $y_{il}$ and $r_{il}$:

|  | $P\left( y_{i} \vert r_{i},\pi_{i1},\pi_{i2} \right)=\sum_{y_{i1}=0}^{min(r_{i1},y_{i})} \sum_{r_{i1}=0}^{r_{i}} P(y_{i1}\vert r_{i1},\pi_{i1})P(y_{i}-y_{i1}\vert r_{i}-r_{i1},\pi_{i2})P(r_{i1}\vert r_{i}),$ | (1) |
| --- | --- | --- |

where $P(y_{i1}|r_{i1},\pi_{i1})$ follows a binomial distribution $y_{i1}\sim Bin(r_{i1},\pi_{i1})$; $P(y_{i}-y_{i1}|r_{i}-r_{i1},\pi_{i2})$ follows a binomial distribution $y_{i}-y_{i1}\sim Bin(r_{i}-r_{i1},\pi_{i2})$; and $P\left( r_{i1} | r_{i} \right)$ follows a binomial distribution $r_{i1}\sim Bin(r_{i},0.5)$. Here, $y_{i1}$ and $y_{i2}$ are conditionally independent given $\pi_{i1}$ and $\pi_{i2}$, though $\pi_{i1}$ and $\pi_{i2}$ are correlated with each other. To ensure scalable computation, we approximate the above $P\left( y_{i} | r_{i},\pi_{i1},\pi_{i2} \right)$ using a binomial mixed model in the following simple form:

$$y_{i}\sim Bin\left( r_{i},\tilde{\pi}_{i} \right), (2)$$

where $\tilde{\pi}_{i}$ is a mean that depends on $\pi_{i1}$ and $\pi_{i2}$, and thus is a function of ($\mu,\beta,{x_{i1},x_{i2},g}_{i},u_{i},e_{i1},e_{i2}$). We obtain an estimate of $\tilde{\pi}_{i}$ by matching the first moment in the likelihood function defined by equation (2) to the first moment in the likelihood function defined by equation (1). Specifically, the expectation of $y_{i}$ under equation (1) can be expressed as:

$$E\left( y_{i} \right)=E\left( y_{i1}+y_{i2} \right)=E\left( E\left( y_{i1}+y_{i2}|r_{i1},r_{i2} \right) \right)=r_{i}\frac{\pi_{i1}+\pi_{i2}}{2} .$$

Therefore, by matching moments, we have

$$\tilde{\pi}_{i}=(\pi_{i1}+\pi_{i2})/2$$

$$=(g^{-1}\left( \mu+x_{i1}\beta+g_{i}+{u_{i}+e}_{i1} \right)+g^{-1}\left( \mu+x_{i2}\beta+g_{i}+{u_{i}+e}_{i2} \right))/2, (3)$$

with

$$(\begin{matrix} {u_{i}+e}_{i1} \\ {u_{i}+e}_{i2} \end{matrix})\sim N(0,{(\sigma}_{u}^{2}+\sigma_{e}^{2})\left( \begin{matrix} 1 & \rho\\ \rho& 1 \end{matrix} \right)), \rho=\frac{\sigma_{u}^{2}}{\sigma_{u}^{2}+\sigma_{e}^{2}},$$

where $\mu$ is the intercept;$x_{il}$ is the *l*th allele type for *i*th individual for the SNP of interest ($x_{il}=0$ or 1, corresponding to the reference allele and alternative allele, respectively); $\beta$ is the corresponding allele/genotype effect size; $g_{i}$ represents the genetic background/polygenic effect of *i*th individual; $u_{i}$ represents the common individual-level environmental effect that varies across individuals but is shared between the two alleles within the same individual;$e_{il}$ represents the residual error and is used to account for independent environmental noise that varies across both individuals and alleles; $g(\cdot)$ denotes the logit link function.

Because we focus on homozygous individuals where $x_{i1}=x_{i2}=x_{i}$, we denote $\lambda_{0}=\mu+x_{i}\beta+g_{i}+u_{i}+(e_{i1}+e_{i2})/2$, which is the average of $\mu+x_{i1}\beta+g_{i}+{u_{i}+e}_{i1}$ and $\mu+x_{i2}\beta+g_{i}+{u_{i}+e}_{i2}$. Due to the $g^{-1}$ function, equation (3) is not in an easy-to-evaluate form. Instead, we further approximate each term in equation (3) through a first order Taylor expansion at $\lambda_{0}$:

$$g^{-1}\left( \mu+x_{i1}\beta+g_{i}+{u_{i}+e}_{i1} \right)\approx\frac{e^{\lambda_{0}}}{e^{\lambda_{0}}+1}\left( 1+\frac{\frac{e_{i1}-e_{i2}}{2}}{e^{\lambda_{0}}+1} \right),$$

$$g^{-1}\left( \mu+x_{i1}\beta+g_{i}+{u_{i}+e}_{i2} \right)\approx\frac{e^{\lambda_{0}}}{e^{\lambda_{0}}+1}\left( 1+\frac{\frac{e_{i2}-e_{i1}}{2}}{e^{\lambda_{0}}+1} \right).$$

Therefore, we have

$$\tilde{\pi}_{i}\approx\frac{e^{\lambda_{0}}}{e^{\lambda_{0}}+1},$$

or equivalently

$$g\left( \tilde{\pi}_{i} \right)\approx\lambda_{0}=\mu+x\beta+g_{i}+u_{i}+\frac{e_{i1}+e_{i2}}{2} (5)$$

for homozygous individuals. Note the above equation (5) holds exactly for heterozygous individuals, since we have $g\left( \pi_{il} \right)=\mu+x_{il}\beta+g_{i}+u_{i}+e_{il}$ for each allele of the heterozygous individuals (main text equation (2)). Subsequently, we can define an approximate background heritability as

$$h^{2}=\frac{\sigma_{g}^{2}}{\sigma_{g}^{2}+\sigma_{u}^{2}+\sigma_{e}^{2}/2}. (6)$$

**2. IMAGE Inference Algorithm**

For homozygous individual $i$, if we denote the random effects for individual $i$ as $b_{i}=g_{i}+u_{i}+\frac{e_{i1}+e_{i2}}{2}$, we have:

| $y_{i}\sim Bin\left( r_{i},\tilde{\pi}_{i} \right), \mathrm{logit}\left( \tilde{\pi}_{i} \right)=\mu+x_{i}\beta+b_{i}.$ | $(7)$ |
| --- | --- |

For heterozygous individual $i$, the corresponding model is as follows:

| $y_{il}\sim Bin\left( r_{il},\pi_{il} \right), \mathrm{logit}\left( \pi_{il} \right)=\mu+x_{il}\beta+b_{il},l=1,2.$ | $(8)$ |
| --- | --- |
|  |  |

We treat the data for a homozygous individual, $\left( y_{i},r_{i} \right)$, as one observation. We treat the data for a heterozygous individual $\left( y_{i1},r_{i1},y_{i2},r_{i2} \right)$as two observations $(\left( y_{i1},r_{i1} \right),\left( y_{i2},r_{i2} \right)$). Given $n_{1}$ homozygous individuals (both types of homozygotes) and $n_{2}$ heterozygous individuals, we have$n^{'}=n_{1}+2n_{2}$ observations in total. To facilitate algebra derivation, we ordered observations by listing homozygous observations first, followed by heterozygous observations. The first and second moments in the likelihood functions defined in equations (7) and (8) can be written in vector and matrix forms as:

|  | $E\left( g\left( \boldsymbol{\pi} \right) \right)=\mu+\boldsymbol{x}\beta,$  $V\left( g\left( \pi\right) \right)=\sigma_{g}^{2}\left( \begin{matrix} \boldsymbol{K}_{n_{1}n_{1}} & \boldsymbol{K}_{n_{1}n_{2}}\otimes\begin{matrix} [1 & 1] \end{matrix} \\ \boldsymbol{K}_{n_{2}n_{1}}\otimes\left[ \begin{matrix} 1 \\ 1 \end{matrix} \right] & \boldsymbol{K}_{n_{2}n_{2}}\otimes\left[ \begin{matrix} 1 & 1 \\ 1 & 1 \end{matrix} \right] \end{matrix} \right){+\sigma_{u}^{2}\left( \begin{matrix} \boldsymbol{I}_{n_{1}\times n_{1}} & \boldsymbol{0}_{n_{1}\times n_{2}} \\ \boldsymbol{0}_{n_{2}\times n_{1}} & \boldsymbol{I}_{n_{2}\times n_{2}}\otimes[\begin{matrix} 1 & 1 \\ 1 & 1 \end{matrix}] \end{matrix} \right)+\sigma}_{e}^{2}\left( \begin{matrix} \frac{1}{2}\boldsymbol{I}_{n_{1}\times n_{1}} & \boldsymbol{0}_{n_{1}\times n_{2}} \\ \boldsymbol{0}_{n_{2}\times n_{1}} & \boldsymbol{I}_{n_{2}\times n_{2}}\otimes[\begin{matrix} 1 & 0 \\ 0 & 1 \end{matrix}] \end{matrix} \right),$ | $(9)$ |
| --- | --- | --- |

where both $g\left( \boldsymbol{\pi} \right)$ and $\boldsymbol{x}$ are $n^{'}$ dimensional vectors by stacking the corresponding values from homozygous observations and heterozygous observations; $\boldsymbol{K}_{n_{1}n_{1}}$, $\boldsymbol{K}_{n_{2}n_{2}}$, and$\boldsymbol{K}_{n_{1}n_{2}}$ are the corresponding kinship matrices computed among homozygous observations, among heterozygous observations, and between homozygous and heterozygous observations, respectively; and $\otimes$ denotes a Kronecker product.

We denote $\boldsymbol{b=g+u+e}$, as the $n^{'}$-vector of random effects. Our likelihood for all observations requires integrating out the random effects and thus is in a form of an n-dimensional integration

$$L=\int\prod_{i=1}^{n^{'}} f(\boldsymbol{y}_{\boldsymbol{i}}|\mu, \beta,\mathbf{b})f\left( \mathbf{b} | \sigma_{g}^{2},\sigma_{e}^{2}, \rho\right)d\boldsymbol{b}.$$

We develop an algorithm based on the penalized quasilikelihood (PQL) [[1](#_ENREF_1)] for parameter inference for IMAGE. Briefly, PQL employs an iterative numerical optimization procedure. In each iteration, we introduce a set of pseudo-data $\tilde{\boldsymbol{y}}$ to replace the originally observed count data $\boldsymbol{y}$. The pseudo-data $\tilde{\boldsymbol{y}}$ is obtained based on a second order Taylor expansion using the conditional distribution $P(y_{i}|\mu,\beta,\boldsymbol{b})$ using the first and second order moments $E(y_{i}|\mu,\beta,\boldsymbol{b})$ and $V(y_{i}|\mu,\beta,\boldsymbol{b})$, both evaluated at the current estimates of the fixed coefficients as well as the random effects $\boldsymbol{b}$. With the pseudo-data, the complex likelihood function for the original data $\boldsymbol{y}$ is replaced by a much simpler linear mixed model (LMM) likelihood function for the pseudo-data $\tilde{\boldsymbol{y}}$, thereby alleviating much of the computational burden associated with our count model. With pseudo-data $\tilde{\boldsymbol{y}}$, we can perform inference and update parameters using the standard average information (AI) algorithm for LMMs [[2-4](#_ENREF_2)]. By iterating between the approximation step of obtaining the pseudo-data $\tilde{\boldsymbol{y}}$ and the inference step of updating the parameter estimates via the AI algorithm, PQL allows us to perform inference in a computationally efficient fashion. Below, we describe the estimation and inference procedure in detail.

The observations $\left( y_{i},r_{i} \right) (i=1,2,\ldots,n^{'}; n^{'}=n_{1}+2n_{2}$; note our slight abuse of notation here) are independent conditional on the unobserved random effects $\boldsymbol{b}$ and the fixed effects $\mu+\boldsymbol{x}\beta,$ with conditional mean $E\left( y_{i} | \mu,\beta,\boldsymbol{b} \right)\mathbf{=}\pi_{i}r_{i}=g^{-1}(\mu+x_{i}\beta+b_{i})$ and conditional variance $V\left( y_{i} | \mu,\beta,\boldsymbol{b} \right)={\nu(\pi_{i})=\pi}_{i}(1-\pi_{i})r_{i}$. We use these two conditional moments to obtain the quasi-likelihood for $ith$ observation as $ql_{i}\left( \mu, \beta|\boldsymbol{b} \right)=\int_{y_{i}}^{\pi_{i}r_{i}} \frac{y_{i}-t}{\nu(t)}dt$, which serve as an approximation for the conditional likelihood. The joint likelihood function can be approximated by the joint quasi-likelihood function

$$ql\left( \mu,\beta,{\sigma_{g}^{2},\sigma}_{u}^{2},\sigma_{e}^{2} \right)=log\int\left( \prod_{i=1}^{n} ql_{i}\left( \boldsymbol{b,}\mu, \beta\right) \right)P\left( \boldsymbol{b|}{\sigma_{g}^{2},\sigma}_{u}^{2},\sigma_{e}^{2} \right)d\boldsymbol{b}.$$

We use Laplace approximation to further approximate the above function and obtain:

|  | $\tilde{ql}\left( \mu,\beta,{\sigma_{g}^{2},\sigma}_{u}^{2},\sigma_{e}^{2} \right)=\frac{1}{2}\log\left\vert\mathbf{VD}+\mathbf{I} \right\vert+\sum_{i=1}^{n} {ql}_{i}\left( \tilde{\boldsymbol{b}}, \mu,\beta\right)-\frac{1}{2}{\tilde{\boldsymbol{b}}}^{T}\mathbf{V}^{-1}\tilde{\boldsymbol{b}},$ | $(10)$ |
| --- | --- | --- |

where$\mathbf{V}=\sigma_{g}^{2}\boldsymbol{V}_{\mathbf{1}}+\sigma_{u}^{2}\boldsymbol{V}_{\mathbf{2}}+\sigma_{e}^{2}\boldsymbol{V}_{\mathbf{3}}$,$\tilde{\boldsymbol{u}}=\underset{\boldsymbol{u}}{\mathrm{argmax}} \left( \sum_{i=1}^{n} {ql}_{i}\left( \left. \mu,\beta\right|\boldsymbol{b} \right)-\frac{1}{2}\boldsymbol{b}^{T}\mathbf{V}^{-1}\boldsymbol{b} \right)$ and $\mathbf{D}=diag\left( 1/{g'\left( \lambda_{i} \right)} \right)$ is a diagonal weight matrix. $\boldsymbol{V}_{\mathbf{1}}=\left( \begin{matrix} K_{n_{1}n_{1}} & K_{n_{1}n_{2}}\otimes\begin{matrix} [1 & 1] \end{matrix} \\ K_{n_{2}n_{1}}\otimes\left[ \begin{matrix} 1 \\ 1 \end{matrix} \right] & K_{n_{2}n_{2}}\otimes\left[ \begin{matrix} 1 & 1 \\ 1 & 1 \end{matrix} \right] \end{matrix} \right)$, $\boldsymbol{V}_{\mathbf{2}}=\left( \begin{matrix} \boldsymbol{I}_{n_{1}\times n_{1}} & \mathbf{0}_{n_{1}\times n_{2}} \\ \mathbf{0}_{n_{2}\times n_{1}} & \boldsymbol{I}_{n_{2}\times n_{2}}\otimes[\begin{matrix} 1 & 1 \\ 1 & 1 \end{matrix}] \end{matrix} \right)$,$\boldsymbol{V}_{\mathbf{3}}=\left( \begin{matrix} \frac{1}{2}\boldsymbol{I}_{n_{1}\times n_{1}} & \mathbf{0}_{n_{1}\times n_{2}} \\ \mathbf{0}_{n_{2}\times n_{1}} & \boldsymbol{I}_{n_{2}\times n_{2}}\otimes[\begin{matrix} 1 & 0 \\ 0 & 1 \end{matrix}] \end{matrix} \right)$.

We treat the approximated quasi-likelihood function $\tilde{ql}$ in equation (10) as the target function. And we obtain estimates for $(\boldsymbol{b}, \mu, \beta)$ and (${\sigma_{g}^{2},\sigma}_{u}^{2},\sigma_{e}^{2}$) alternately from equation (10).

Specifically, we first obtain estimates for $(\boldsymbol{b}, \mu, \beta)$ conditional on the current estimates of (${\sigma_{g}^{2},\sigma}_{u}^{2},\sigma_{e}^{2}$). To do so, following [[1](#_ENREF_1)], we assume that the iterative weights vary slowly with respect to the conditional mean; that is

$$\frac{\partial\mathbf{D}}{\partial\lambda_{i}}\approx0.$$

We then obtain the first order derivatives with respect to either $(\mu, \beta)$ or $\boldsymbol{b}$, and set the two first order derivatives to zero; that is

|  | $\left( \mathbf{1},\boldsymbol{x} \right)^{T}\mathbf{D}\Delta\left( \boldsymbol{y}-\boldsymbol{\lambda r} \right)=\mathbf{0},$ | $(11)$ |
| --- | --- | --- |
|  | $\mathbf{D}\Delta\left( \boldsymbol{y}-\boldsymbol{\lambda r} \right)-\mathbf{V}^{-\mathbf{1}}\boldsymbol{b}=\mathbf{0},$ | $(12)$ |

where $\boldsymbol{\lambda}=(\lambda_{1}, \cdots,\lambda_{n_{1}+2n_{2}})$ and $\Delta=diag\left\{ g'\left( \lambda_{i} \right) \right\}$.

We now define the pseudo-data

|  | $\tilde{y_{i}}=\eta_{i}+g^{'}\left( \lambda_{i} \right)\left( y_{i}-\lambda_{i}r_{i} \right),$ | $(13)$ |
| --- | --- | --- |

and our equation (11) becomes

|  | $\boldsymbol{b}=\mathbf{V}\mathbf{H}^{-1}\left[ \tilde{\boldsymbol{y}}-\left( \mathbf{1},\boldsymbol{x} \right)\left( \begin{matrix} \mu\\ \boldsymbol{\beta} \end{matrix} \right) \right],$ | $(14)$ |
| --- | --- | --- |

where $\mathbf{H}=\mathbf{D}^{-1}+\mathbf{V}$. Substituting equation (14) into equation (12), we can obtain the estimates

|  | $\left( \begin{matrix} \hat{\mu} \\ \hat{\boldsymbol{\beta}} \end{matrix} \right)= \left[ \left( \mathbf{1},\boldsymbol{x} \right)^{T}\mathbf{H}^{-1}\left( \mathbf{1},\boldsymbol{x} \right) \right]^{-1}\left( \mathbf{1},\boldsymbol{x} \right)^{T}\boldsymbol{H}^{-1}\tilde{\boldsymbol{y}},$ | $(15)$ |
| --- | --- | --- |

and

|  | $\hat{\mathbf{b}}=\mathbf{V}\mathbf{H}^{-1}\left[ \tilde{\boldsymbol{y}}-\left( \mathbf{1},\boldsymbol{x} \right)\left( \begin{matrix} \hat{\mu} \\ \hat{\boldsymbol{\beta}} \end{matrix} \right) \right],$ | $(16)$ |
| --- | --- | --- |

Both are conditional on the variance component estimates (${\sigma_{g}^{2},\sigma}_{u}^{2},\sigma_{e}^{2}$).

Next, we obtain estimates for the variance components (${\sigma_{g}^{2},\sigma}_{u}^{2},\sigma_{e}^{2}$) conditional on the current estimates of $(\boldsymbol{b}, \mu, \beta)$*.* Here we first define the variance components $\tau_{1}=\sigma_{g}^{2}$,$\tau_{2}=\sigma_{u}^{2}$ and $\tau_{3}=\sigma_{g}^{2}$. We then integrate out the fixed effects $\mu$ and $\beta$ in equation (10) to obtain the restricted likelihood function as

$$\tilde{ql}_{R}\left( \tau_{1},\tau_{2},\tau_{3} \right)=c_{R}-\frac{1}{2}\log\left| \mathbf{H} \right|-\frac{1}{2}\log\left| \left( \mathbf{1},\boldsymbol{x} \right)^{T}\mathbf{H}^{-1}\left( \mathbf{1},\boldsymbol{x} \right) \right|-\frac{1}{2}{\tilde{\boldsymbol{y}}}^{T}\mathbf{P}\tilde{\boldsymbol{y}},$$

where $\mathbf{P}= \mathbf{H}^{-1}-\mathbf{H}^{-1}\left( \mathbf{1},\boldsymbol{x} \right)^{T}\left( \left( \mathbf{1},\boldsymbol{x} \right)^{T}\mathbf{H}^{-1}\left( \mathbf{1},\boldsymbol{x} \right) \right)^{-1}\left( \mathbf{1},\boldsymbol{x} \right)\mathbf{H}^{-1}$, and $c_{R}$ is a constant. We use the AI algorithm to obtain variance component estimates. In particular, we obtain the first derivatives as

$$\frac{\partial\tilde{ql}_{R}\left( {\sigma_{g}^{2},\sigma}_{u}^{2},\sigma_{e}^{2} \right)}{\partial\tau_{i}}=\frac{1}{2}\left\{ {\tilde{\boldsymbol{y}}}^{T}\mathbf{P}\boldsymbol{V}_{\boldsymbol{i}}\mathbf{P}\tilde{\boldsymbol{y}}-tr\left( \mathbf{P}\boldsymbol{V}_{\boldsymbol{i}} \right) \right\}, i\in\left\{ 1,2,3 \right\},$$

and the second derivatives as

$$\frac{\partial^{2}\tilde{ql}_{R}\left( {\sigma_{g}^{2},\sigma}_{u}^{2},\sigma_{e}^{2} \right)}{\partial\tau_{i}\partial\tau_{j}}=\frac{1}{2}tr\left( \boldsymbol{V}_{\boldsymbol{i}}\mathbf{P}\boldsymbol{V}_{\boldsymbol{j}}\mathbf{P} \right)-{\tilde{\boldsymbol{y}}}^{T}\mathbf{P}\boldsymbol{V}_{\boldsymbol{i}}\mathbf{P}\boldsymbol{V}_{\boldsymbol{j}}\mathbf{P}\tilde{\boldsymbol{y}}; i,j\in\{1,2,3\}$$

The second derivatives constitute the observed information matrix. Because the elements in the expected information matrix are

$$E\left[ \frac{\partial^{2}\tilde{ql}_{R}\left( {\sigma_{g}^{2},\sigma}_{u}^{2},\sigma_{e}^{2} \right)}{\partial\tau_{i}\partial\tau_{j}} \right]=-\frac{1}{2}tr\left( \boldsymbol{V}_{\boldsymbol{i}}\mathbf{P}\boldsymbol{V}_{\boldsymbol{j}}\mathbf{P} \right); i,j\in\left\{ 1,2,3 \right\},$$

we can obtain the average information (AI) matrix as an average of the above two matrices; that is

$$\mathbf{AI=}\frac{\boldsymbol{1}}{\boldsymbol{2}}\left[ \begin{matrix} {\tilde{\boldsymbol{y}}}^{T}\mathbf{P}\boldsymbol{V}_{\boldsymbol{1}}\mathbf{P}\boldsymbol{V}_{\boldsymbol{1}}\mathbf{P}\tilde{\boldsymbol{y}} & {\tilde{\boldsymbol{y}}}^{T}\mathbf{P}\boldsymbol{V}_{\boldsymbol{1}}\mathbf{P}\boldsymbol{V}_{\boldsymbol{2}}\mathbf{P}\tilde{\boldsymbol{y}} & {\tilde{\boldsymbol{y}}}^{T}\mathbf{P}\boldsymbol{V}_{\boldsymbol{1}}\mathbf{P}\boldsymbol{V}_{\boldsymbol{3}}\mathbf{P}\tilde{\boldsymbol{y}} \\ {\tilde{\boldsymbol{y}}}^{T}\mathbf{P}\boldsymbol{V}_{\boldsymbol{2}}\mathbf{P}\boldsymbol{V}_{\boldsymbol{1}}\mathbf{P}\tilde{\boldsymbol{y}} & {\tilde{\boldsymbol{y}}}^{T}\mathbf{P}\boldsymbol{V}_{\boldsymbol{2}}\mathbf{P}\boldsymbol{V}_{\boldsymbol{2}}\mathbf{P}\tilde{\boldsymbol{y}} & {\tilde{\boldsymbol{y}}}^{T}\mathbf{P}\boldsymbol{V}_{\boldsymbol{2}}\mathbf{P}\boldsymbol{V}_{\boldsymbol{3}}\mathbf{P}\tilde{\boldsymbol{y}} \\ {\tilde{\boldsymbol{y}}}^{T}\mathbf{P}\boldsymbol{V}_{\boldsymbol{3}}\mathbf{P}\boldsymbol{V}_{\boldsymbol{1}}\mathbf{P}\tilde{\boldsymbol{y}} & {\tilde{\boldsymbol{y}}}^{T}\mathbf{P}\boldsymbol{V}_{\boldsymbol{3}}\mathbf{P}\boldsymbol{V}_{\boldsymbol{2}}\mathbf{P}\tilde{\boldsymbol{y}} & {\tilde{\boldsymbol{y}}}^{T}\mathbf{P}\boldsymbol{V}_{\boldsymbol{3}}\mathbf{P}\boldsymbol{V}_{\boldsymbol{3}}\mathbf{P}\tilde{\boldsymbol{y}} \end{matrix} \right]\boldsymbol{.}$$

With the first and second order derivatives, we can perform Newton-Raphson update with the AI algorithm and obtain estimates for $\left( {\sigma_{g}^{2},\sigma}_{u}^{2},\sigma_{e}^{2} \right)$.

As a summary, we implement the PQL algorithm that consists of the following steps:

1. Initialize the parameters, $\mu^{\left( 0 \right)}\boldsymbol{,}\beta^{\left( 0 \right)}$,$\boldsymbol{\tau}^{\left( 0 \right)}=\left( \tau_{1}^{\left( 0 \right)},\tau_{2}^{\left( 0 \right)},\tau_{3}^{\left( 0 \right)} \right)^{T},$ and obtain the pseudo-data ${\tilde{\boldsymbol{y}}}^{\left( 0 \right)}$ as in equation (12). Set $t=1$.
2. Update $\boldsymbol{\tau}^{\left( t \right)}\boldsymbol{=}\boldsymbol{\tau}^{\left( t-1 \right)}\boldsymbol{+}\mathbf{AI}^{-1}\left( \frac{\partial{ql}_{R}\left( {\sigma_{g}^{2},\sigma}_{u}^{2},\sigma_{e}^{2} \right)}{\partial\boldsymbol{\tau}} \right)$;
3. Update $\mu^{\left( t \right)}\boldsymbol{,}\beta^{\left( t \right)}$ and $\boldsymbol{b}^{\left( t \right)}$ with $\boldsymbol{\tau}^{\left( t \right)}$ and ${\tilde{\boldsymbol{y}}}^{\left( t-1 \right)}$ as in equations (15) and (16);
4. Update ${\tilde{\boldsymbol{y}}}^{\left( t \right)}$ using the $\mu^{\left( t \right)}, \beta^{\left( t \right)}$ and $\boldsymbol{b}^{\left( t \right)}$ as in equation (13);
5. Set $t=t+1$, and repeat steps 2-4 until convergence.

Once we obtain parameter estimates $(\mu,\beta,{\sigma_{g}^{2},\sigma}_{u}^{2},\sigma_{e}^{2})$, we can construct the Wald test based on

$\hat{\beta}=\left( \boldsymbol{x}^{T}\mathbf{P}_{\boldsymbol{c}}\boldsymbol{x} \right)^{-1}\boldsymbol{x}^{T}\mathbf{P}_{\boldsymbol{c}}\tilde{\boldsymbol{y}}$ and $\mathrm{Var}\left( \hat{\beta} \right)=\left( \boldsymbol{x}^{T}\mathbf{P}_{\boldsymbol{c}}\boldsymbol{x} \right)^{-1}$,

where $\boldsymbol{P}_{\boldsymbol{c}}= \mathbf{H}^{-1}-\mathbf{H}^{-1}\mathbf{W}^{T}\left( \mathbf{W}^{T}\mathbf{H}^{-1}\mathbf{W}^{T} \right)^{-1}\mathbf{W}^{T}\mathbf{H}^{-1}$.

Because of the approximation using PQL, when sample size is small (<150), we observe an inflation of p-values in the simulations and real data applications (e.g. the genomic control factor equals 1.21 and 1.12 in the two real data sets). We therefore perform an additional step of genomic control to adjust for p-values in a *post hoc* fashion. Specifically, we normalize $\chi^{2}$ statistics (across all SNP-CpG pairs) by dividing max(1, $\lambda_{gc}$) and then use the normalized $\chi^{2}$ statistics to obtain the final p-values.

**3. M-values Calculation**

For methods that use normalized data, we performed “M” value transformation. To do so, we first divided the number of methylated reads by the number of unmethylated reads. Afterwards, we performed a log2-transformation to obtain the final normalized data. The final normalized data is in the form of $\log_{2} \left( \frac{methylated reads+a}{unmethylated reads+a} \right)$, where a small value $a$ = 0.01 is added to avoid zero values inside the log function.

**REFERENCES**

1. Breslow, N.E. and D.G. Clayton, *Approximate Inference in Generalized Linear Mixed Models.* Journal of the American Statistical Association, 1993. **88**(421): p. 9-25.

2. Chen, H., et al., *Control for Population Structure and Relatedness for Binary Traits in Genetic Association Studies via Logistic Mixed Models.* American Journal of Human Genetics, 2016. **98**(4): p. 653-666.

3. Gilmour, A.R., R. Thompson, and B.R. Cullis, *Average information REML: An efficient algorithm for variance parameter estimation in linear mixed models.* Biometrics, 1995. **51**(4): p. 1440-1450.

4. Yang, J.A., et al., *GCTA: A Tool for Genome-wide Complex Trait Analysis.* American Journal of Human Genetics, 2011. **88**(1): p. 76-82.
