# Supplementary material for: IMAGE: high-powered detection of genetic effects on DNA methylation using integrated methylation QTL mapping and allele-specific analysis
Source: Genome Biol. 2019 Oct 24;20:220. doi: 10.1186/s13059-019-1813-1 (PMC6813132; doi:10.1186/s13059-019-1813-1)
Supplement: Supplementary file 2 — Additional file 2: Supplementary figures on the performance evaluation of IMAGE and on the quality control of the real data applications. [file 13059_2019_1813_MOESM2_ESM.docx]

**Supplementary Figures**


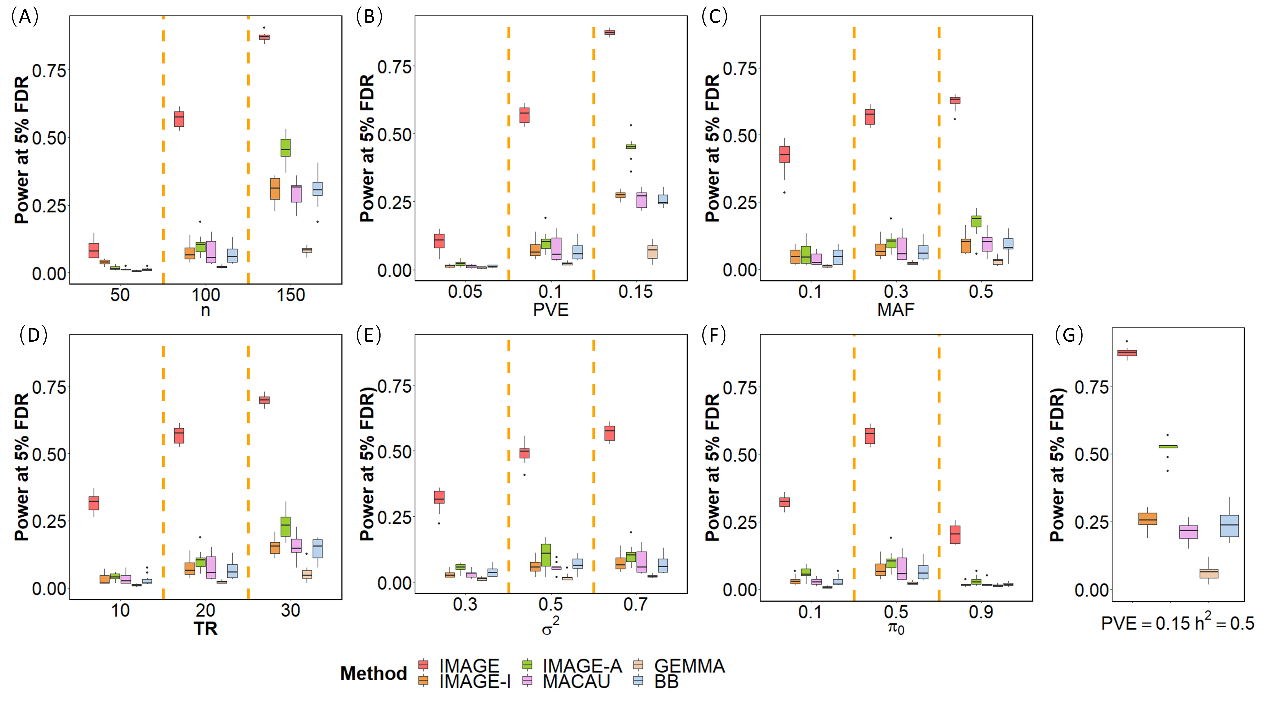


**Fig. S1**: IMAGE achieves higher power to detect mQTL across various simulation settings. Power is measured as the proportion of true positives detected at a false discovery rate (FDR) of 0.05. Each simulation setting consists of 10 simulation replicates, and each replicate includes 10,000 simulated SNP-CpG pairs, with 1,000 mQTL and 9,000 non-mQTL. (A) We vary the sample size $n$ to be either 50, 100, or 150, while maintaining other parameters at the baseline setting. With increasing sample size, all methods gain power. (B) We vary Percentage of Variance Explained by the SNP ($PVE)$ to be either 0.05, 0.1, or 0.15, while maintain other parameters at the baseline setting. With increasing $PVE$, all methods gain power. (C) We vary Minor Allele Frequency ($MAF)$ to be either 0.1, 0.3, or 0.5, while maintaining other parameters at the baseline setting. With increasing $MAF$, all methods gain power. (D) We vary average read counts per site ($TR)$ to be either 10, 20, or 30, while maintaining other parameters at the baseline setting. With increasing $TR$, all methods gain power. (E) We vary over-dispersion variance ($\sigma^{2}$) to be either 0.3,0.5, or 0.7, while maintaining other parameters at the baseline setting. With increasing $\sigma^{2}$, all methods gain power. (F) We vary mean methylation ratio ($\pi_{0}$) to be either 0.1, 0.5, or 0.9, while maintaining other parameters at the baseline setting. The power of all methods is highest for a moderate methylation level $\pi_{0}$, but reduces for hypermethylated and hypomethylated sites. (G) We set $h^{2}=0.5$ and $PVE=0.15$ in the simulations to mimic our real data applications. The power comparison results here are largely consistent with what we saw in the real data. FDR: false discovery rate. The middle panel in (A), (B), (C), (D), (F) and the right panel in (E) correspond to the baseline simulation setting.


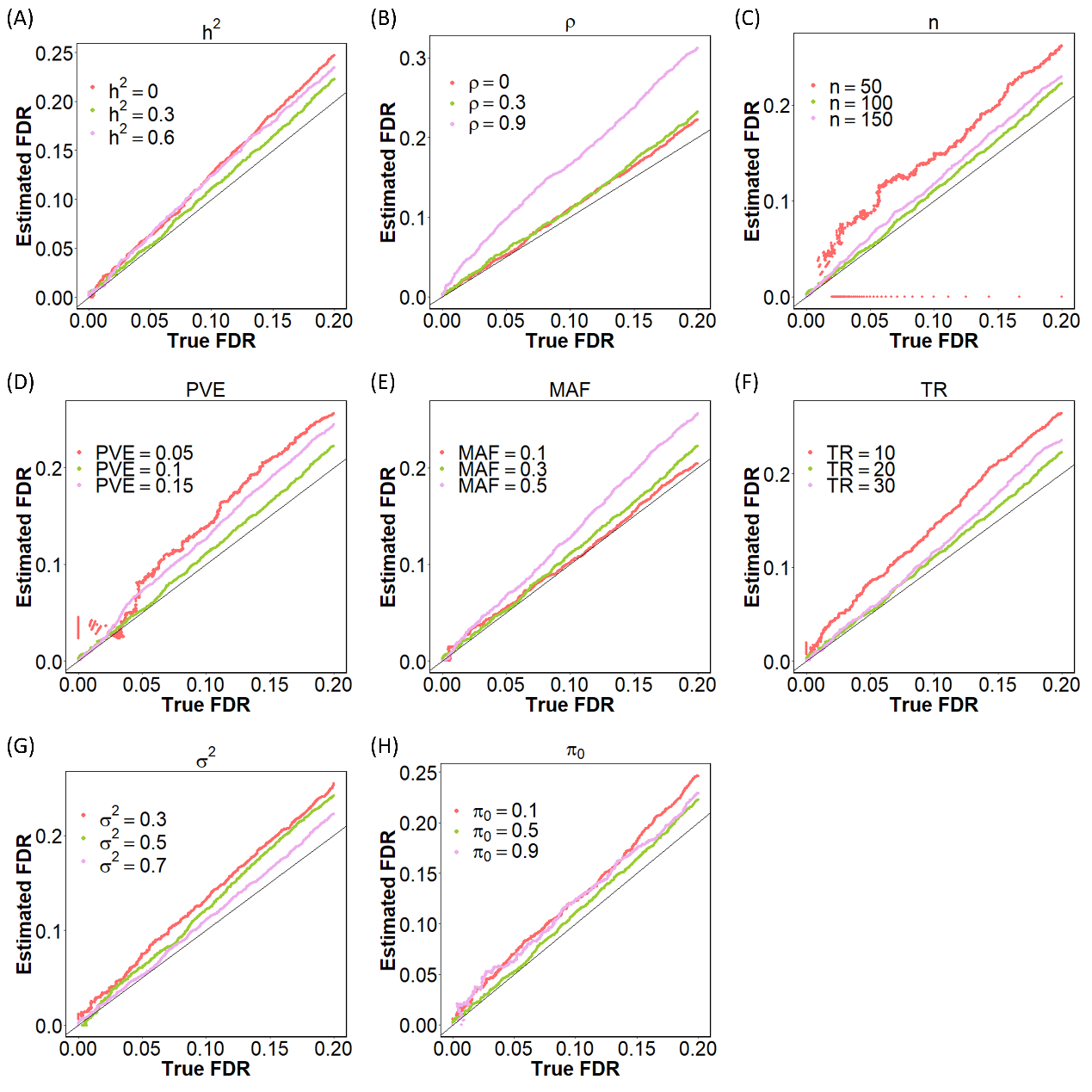


**Fig. S2**: IMAGE yields reasonably well-calibrated false discovery rate (FDR) estimates across a range of parameter values in simulations. Each simulation setting includes 100,000 simulated SNP-CpG pairs, with 10,000 associated pairs and 90,000 unassociated pairs. In each simulation setting, we performed 10 permutations to estimate the empirical FDR (y-axis), which is plotted against the true FDR (x-axis). In (A), we vary $h^{2}$ to be 0 (red), 0.3 (green), or 0.6 (pink). In (B), we vary $\rho$ to be either 0 (red), 0.3 (green), or 0.9 (pink). In (C), we vary $n$ to be either 50 (red), 100 (green), or 150 (pink). In (D), we vary $\mathrm{PVE}$ to be either 0.05 (red), 0.1 (green), or 0.15 (pink). In (E), we vary $\mathrm{MAF}$ to be either 0.05 (red), 0.3 (green), or 0.5 (pink). In (F), we vary $\mathrm{TR}$ to be either 10 (red), 20 (green), or 30 (pink). In (G), we vary $\sigma^{2}$ to be either 0.3 (red), 0.5 (green), or 0.7 (pink). In (H), we vary $\pi_{0}$ to be either 0.1 (red), 0.3 (green), or 0.5 (pink). In each panel, we maintain the other parameters as in the baseline setting. Note that the upward deviation from the x=y line reflects a conservative estimate of the FDR: thus, IMAGE is not likely to generate more false discoveries than expected at a given FDR threshold, although it will miss a small number of likely true positives.


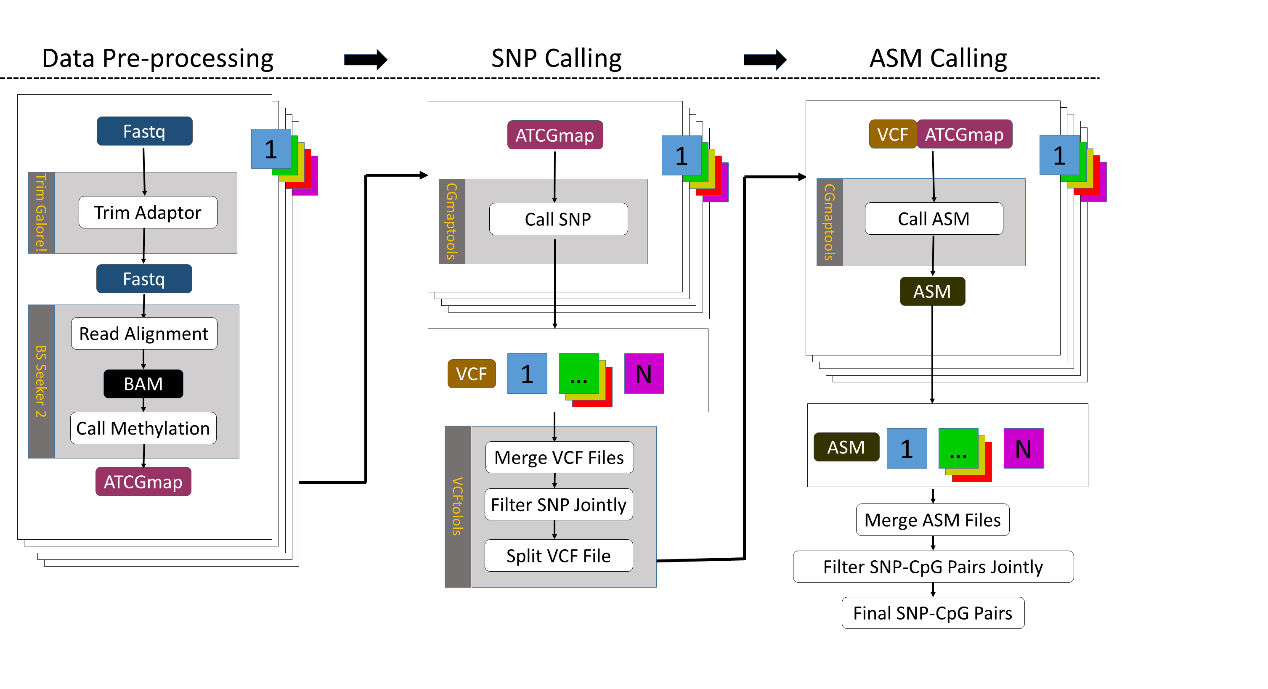


**Fig. S3**: Schematic of the data processing steps for the baboon and wolf data sets. We processed input files in the fastq format to obtain both SNP information and methylation information, which were paired together and subjected through various filtering steps to yield a final set of SNP-CpG pairs for analysis. Briefly, steps include: (1) trimmed the adaptors in the fastq files using TrimGalore!; (2) Mapped to the baboon reference genome *Panu2.0* or the dog reference genome *canFam3.1* using BSseeker2; (3) Called SNPs using CGmaptools with the BayesWC strategy; (4) Merged and filtered VCF files using VCFtools; (5) Called allele-specific methylation using CGmaptools; (6) Merged and filtered ASM data to yield a final set of SNP-CpG pairs. In the above figure, Fastq, BAM, ATCGmap, VCF, and ASM refer to different file formats.


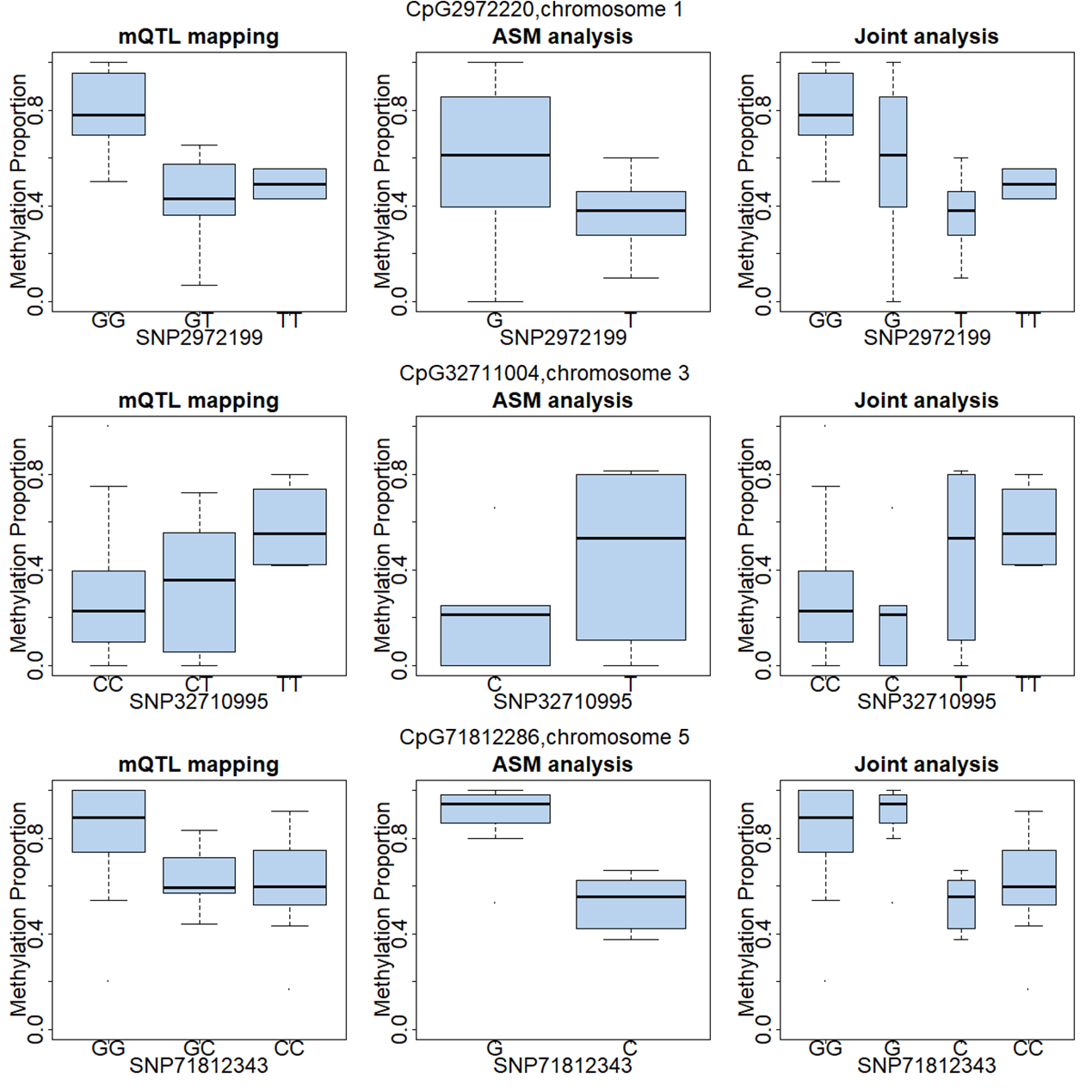


**Fig. S4**: Examples of associated SNP-CpG pairs detected by IMAGE in the baboon data. Each row contains one SNP-CpG pair. Boxplots on the three left panels show individual-level methylation information used by mQTL mapping methods. Boxplots on the three middle panels show the allele-specific methylation information used by ASM analysis methods. Boxplots on the three right panels show both individual-level methylation information and allele-specific methylation information used by IMAGE.


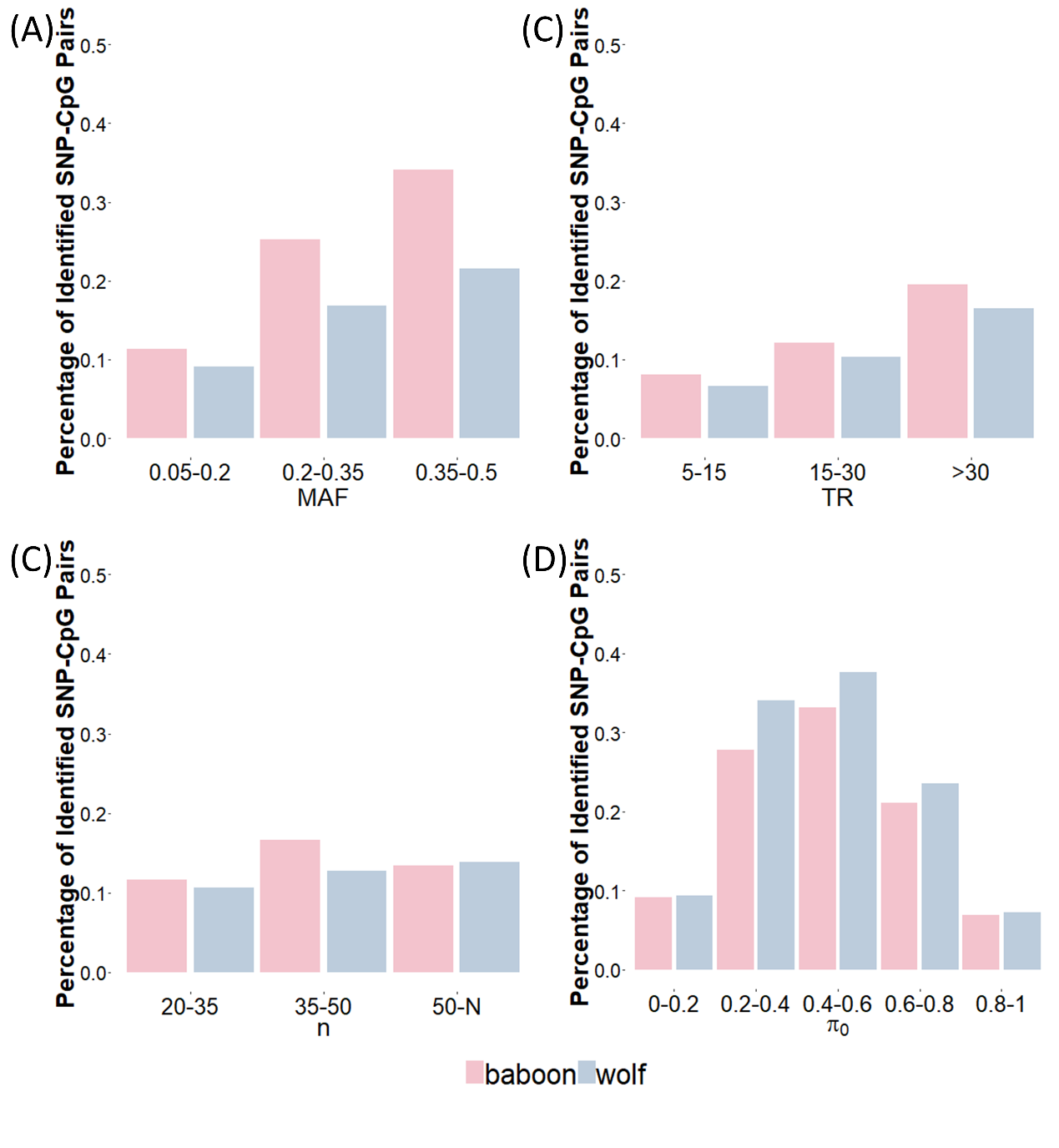


**Fig. S5**: Number of associated SNP-CpG pairs identified by IMAGE for sites stratified by MAF, TF, n, and methylation level in the baboon (pink) and wolf data (blue). IMAGE achieves higher power with increasing MAF (A), increasing read depth (B), increasing sample size (C), and intermediate methylation levels (D). In (C), N represents the sample size (67 for the baboon data and 63 for the wolf data).


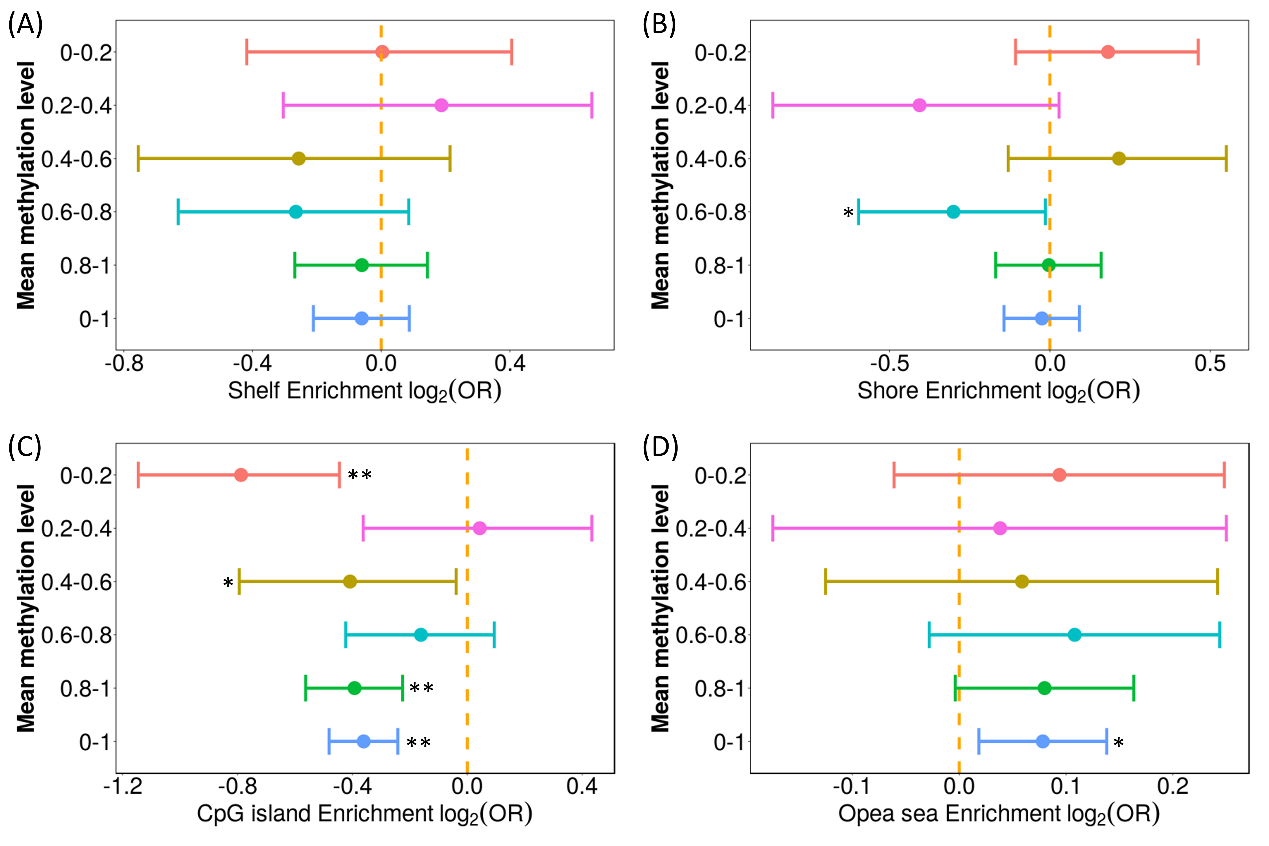


**Fig. S6**: CpG sites with IMAGE-identified mQTL, regardless of methylation levels, are enriched in open sea regions and depleted in CpG islands in the baboon data. log2 odds ratio (x-axis) of detecting associated SNP-CpG pairs, together with the 95% CI, is computed for CpG sites residing in different annotated genomic regions, further stratified with regard to methylation levels (y-axis). CpG sites with IMAGE-identified mQTL are enriched in open sea regions and depleted in CpG islands. *P<0.05. **P<0.01.


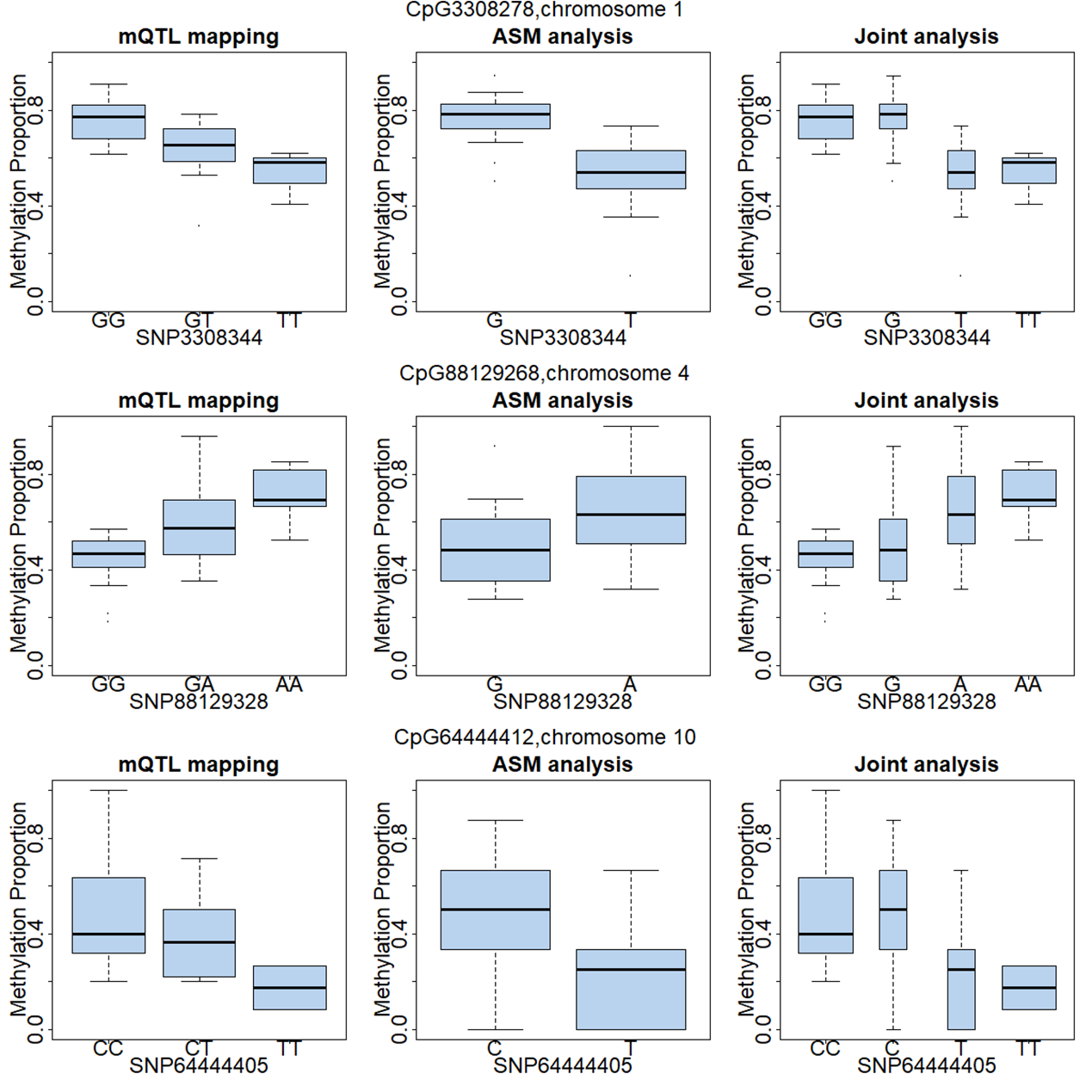


**Fig. S7**: Examples of associated SNP-CpG pairs detected by IMAGE in the wolf data. Each row contains one SNP-CpG pair. Boxplots on the three left panels show individual-level methylation information used by mQTL mapping methods. Boxplots on the three middle panels show the allele-specific methylation information used by ASM analysis methods. Boxplots on the three right panels show both individual-level methylation information and allele-specific methylation information used by IMAGE.


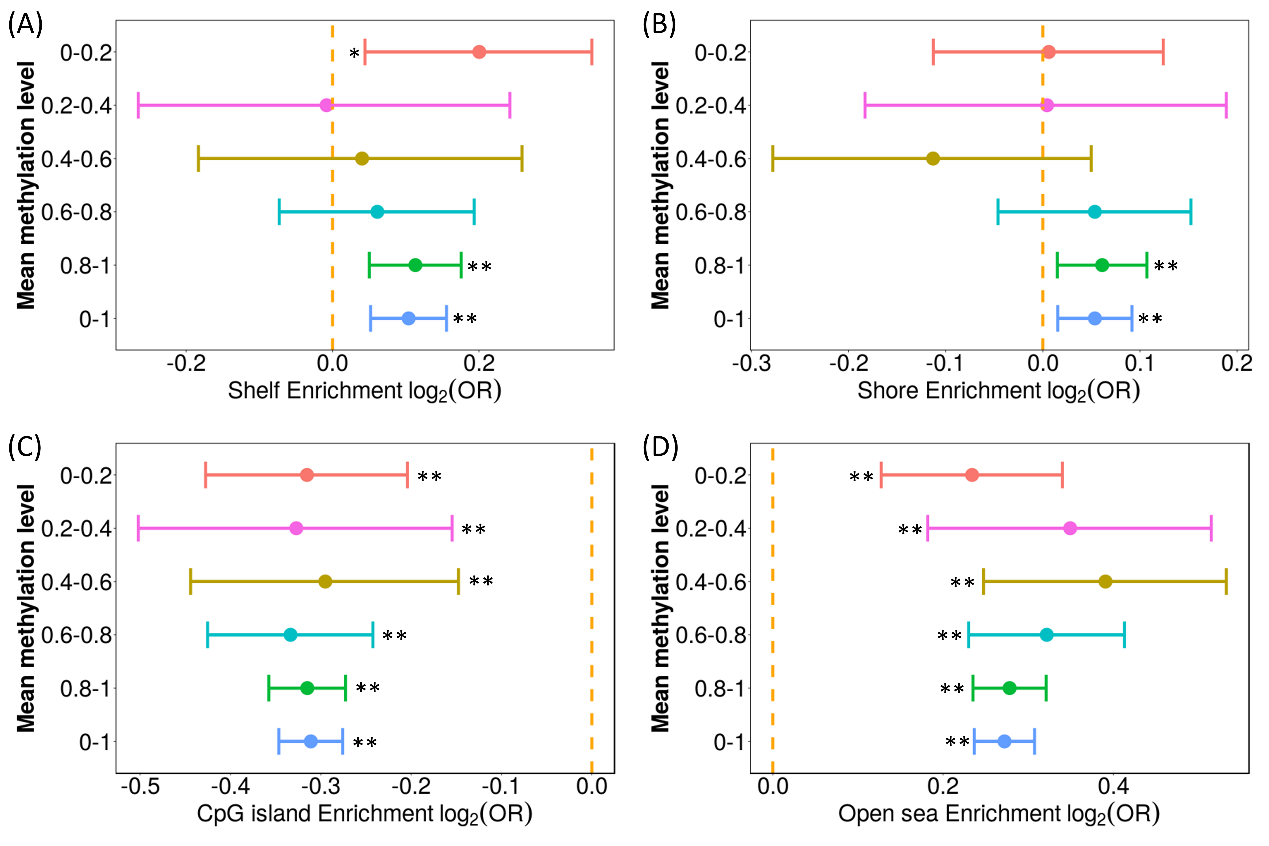


**Fig. S8**: CpG sites with IMAGE-identified mQTL, regardless of methylation levels, are enriched in open sea regions and depleted in CpG islands in the wolf data. log2 odds ratio (x-axis) of detecting associated SNP-CpG pairs, together with the 95% CI, is computed for CpG sites residing in different annotated genomic regions, further stratified with regard to methylation levels (y-axis). CpG sites with IMAGE-identified mQTL are enriched in open sea regions and depleted in CpG islands. *P<0.05. **P<0.01.


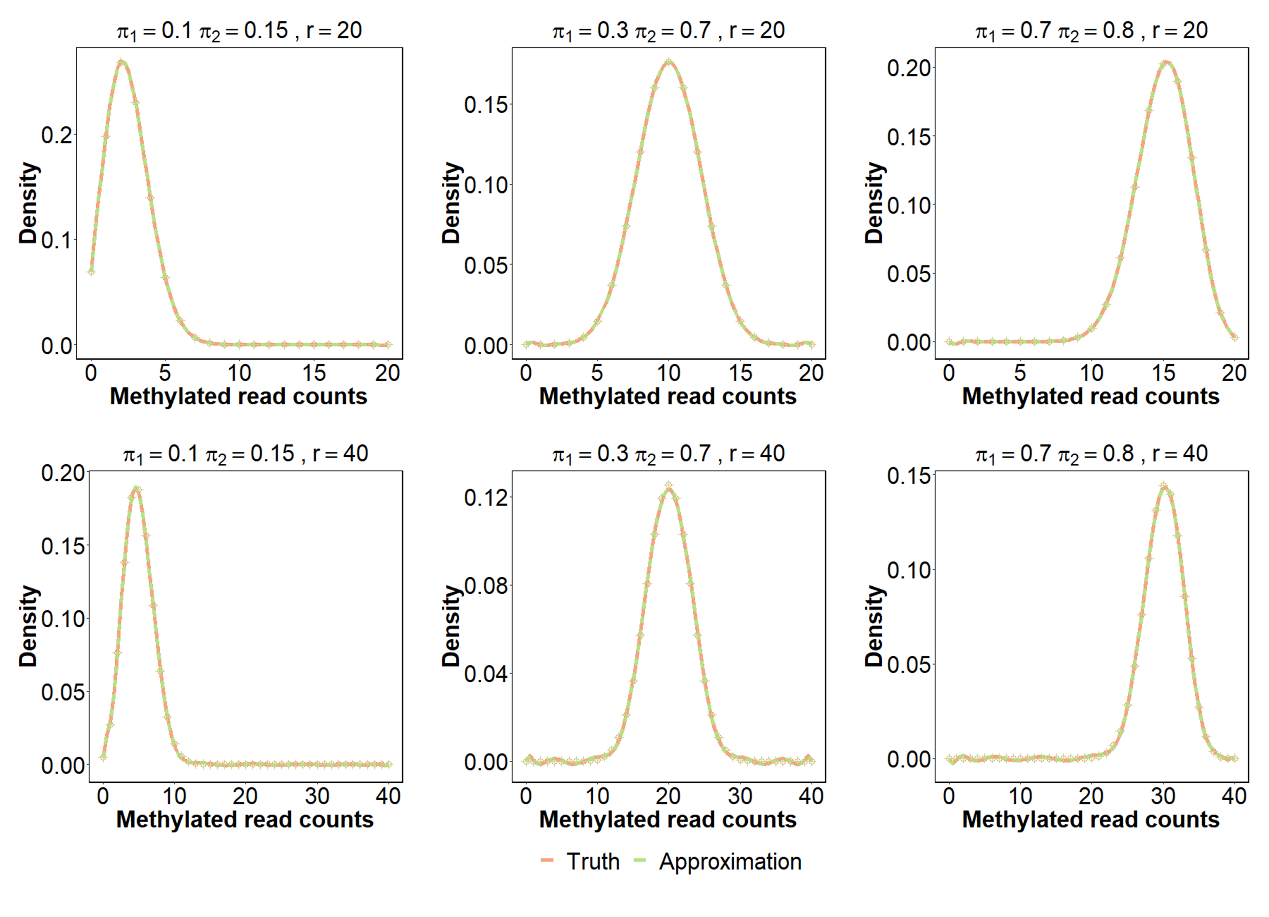


**Fig. S9**: The binomial approximation used in IMAGE through matching moments approximates the true underlying density in a variety of parameter settings. The parameters examined include $\pi_{1}, \pi_{2},r$ (on top of each panel) and the methylated read counts (on x-axis). The approximation works well across a range of settings.


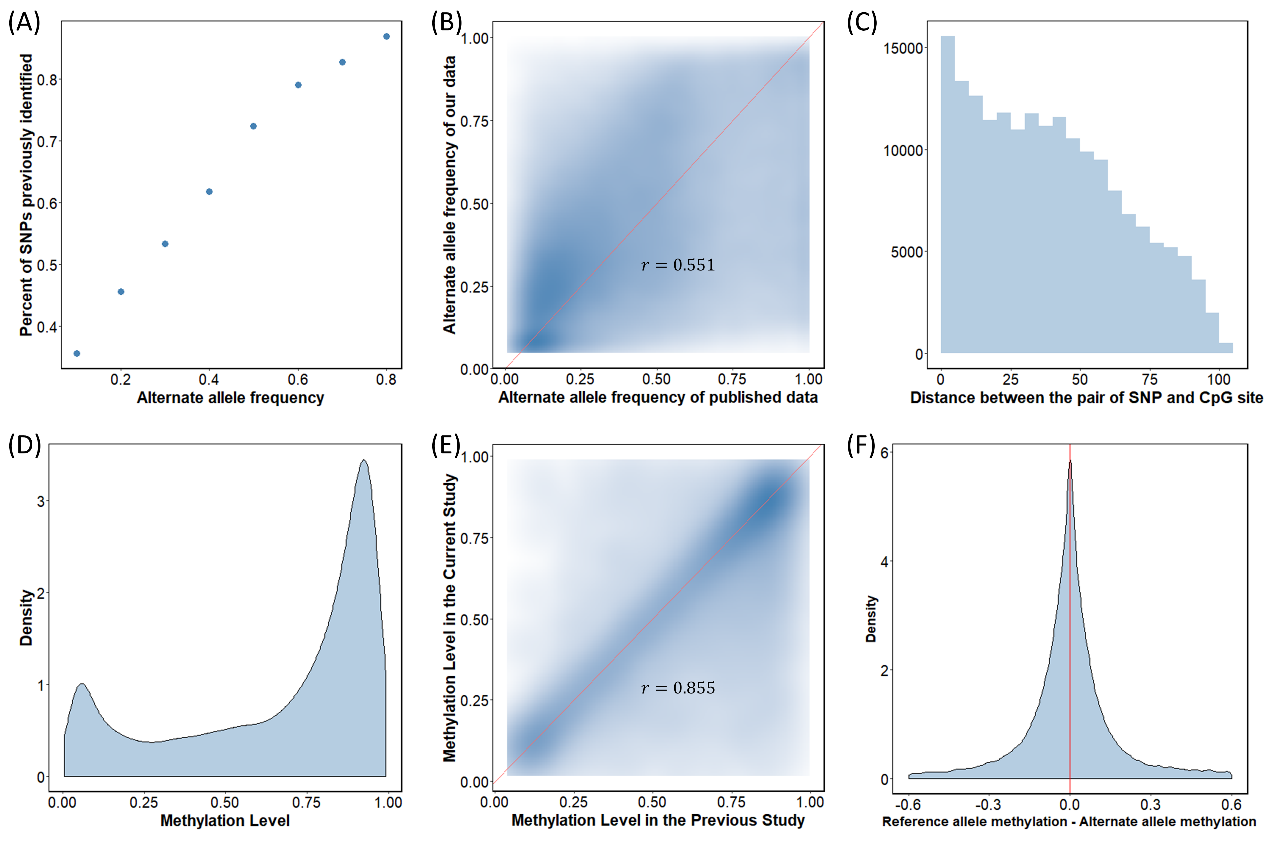


**Fig. S10**: Quality control in the baboon data. (A) Variants called in our dataset overlap with variants identified through DNA sequencing in a separate sample in East African baboons from a previous study [[1](#_ENREF_1)]. The overlap proportion (y-axis) increases with increasing alternate allele frequency (x-axis). (B) Among the variants identified in both our data and the previous DNA sequencing study, the alternate allele frequency in our data (y-axis) is highly correlated with the alternate allele frequency in the DNA sequencing study (x-axis). (C) Distribution of the distance between SNP and CpG site across all analyzed SNP-CpG pairs in the baboon data. Because the data were generated using 100 bp reads, 100 bp is the maximum possible distance in our data set. (D) Density plot for the distribution of methylation levels across analyzed CpG sites in the baboon data. The methylation level is measured as the proportion of methylated reads. A bimodal pattern, with both hypomethylated and hypermethylated CpG sites, is clearly visible. (E) The methylation level in our processing pipeline is highly correlated with that measured through in a different processing pipeline in a previous study using the same baboon data [[2](#_ENREF_2)]. (F) Density plot of the difference in methylation level between the reference allele and the alternate allele across CpG sites in the baboon data. The difference is symmetric, suggesting no apparent reference mapping bias in the data.


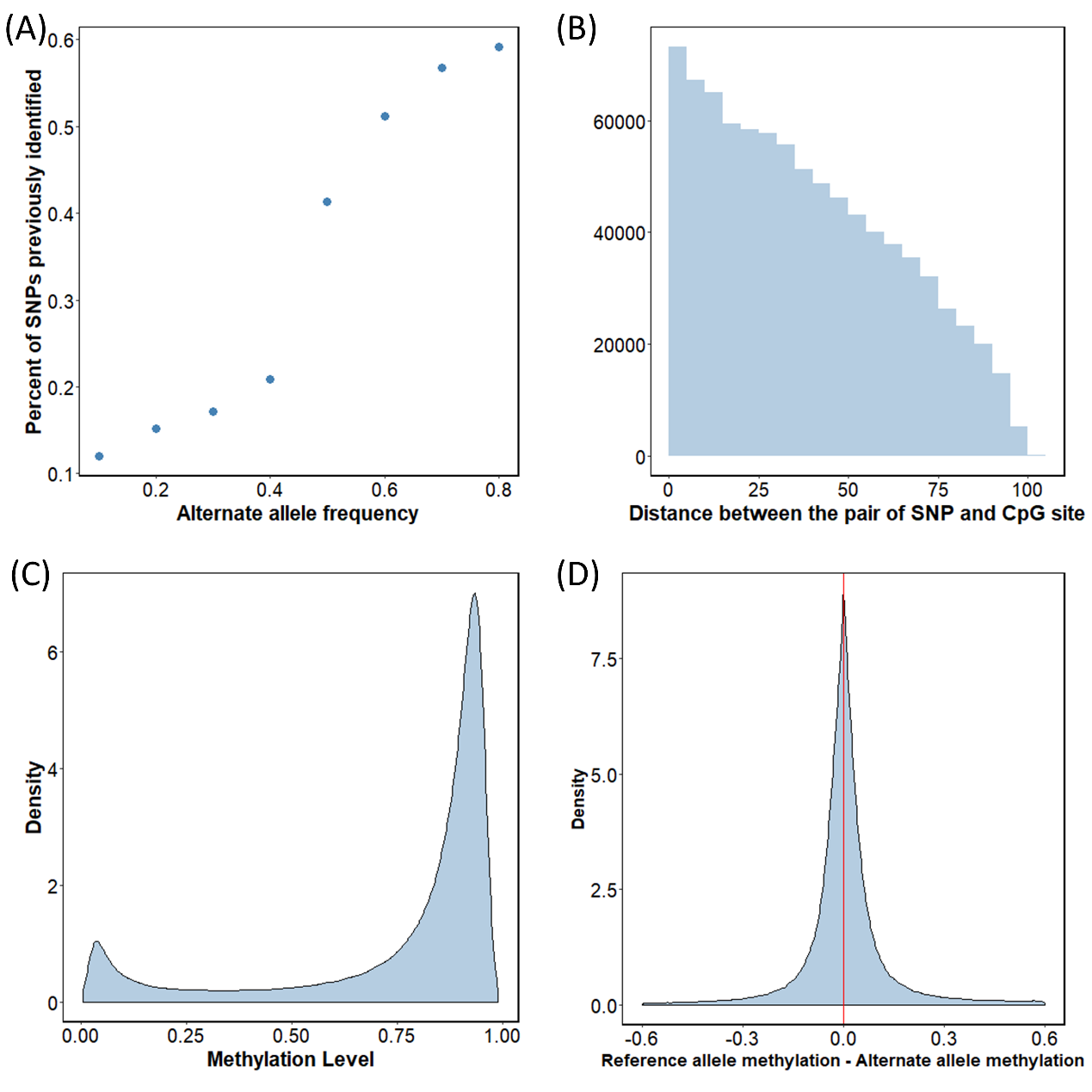


**Fig. S11**: Quality control in the wolf data. (A) Common SNP variants in our wolf dataset are likely to overlap with the variants from the current Ensembl release for dogs. Increasing the alternate allele frequency increases the proportion of SNPs that were previously identified. (B) The histogram of distance between SNPs and CpG sites of the wolf data. Because the data were generated using 100 bp reads, 100 bp is the maximum possible distance in our data set. (C) Density plot of the distribution of the average methylation levels in wolf data. As expected for RRBS data, the data contain a large number of hypomethylated and hypermethylated CpG sites. (D) Density plot of the difference in methylation level between reference and alternate alleles in wolf data. For most CpG sites, there is little or no difference in methylation level between the reference and the alternate alleles.

**REFERENCES**

1. Wall, J.D., et al., *Genomewide ancestry and divergence patterns from low-coverage sequencing data reveal a complex history of admixture in wild baboons.* Molecular Ecology, 2016. **25**(14): p. 3469-3483.

2. Lea, A.J., et al., *Resource base influences genome-wide DNA methylation levels in wild baboons (Papio cynocephalus).* Molecular Ecology, 2016. **25**(8): p. 1681-1696.
