# Supplementary material for: IMAGE: high-powered detection of genetic effects on DNA methylation using integrated methylation QTL mapping and allele-specific analysis
Source: Genome Biol. 2019 Oct 24;20:220. doi: 10.1186/s13059-019-1813-1 (PMC6813132; doi:10.1186/s13059-019-1813-1)
Supplement: Supplementary file 3 — Additional file 3: Supplementary tables on functional enrichment analyses and type I error control examination. [file 13059_2019_1813_MOESM3_ESM.docx]

**Supplementary Tables**

**Table S1**: Enrichment of CpG sites in different genomic regions for CpG sites with mQTL versus CpG sites without mQTL in the baboon data. Percentage of CpG sites in different genomic regions (open sea; shelf; shore; CpG island) for CpG sites with mQTL (3^rd^ column) versus CpG sites without mQTL (4^th^ column). A fold enrichment is computed as the odds ratio (5^th^ column) and tested through a Fisher’s exact test (6^th^ column). mQTL are detected by different methods (IMAGE, IMAGE-I, IMAGE-A, MACAU, GEMMA, BB) based on a permutation-based empirical false discovery rate of 0.05. Bold font highlights significant enrichment (p-value < 0.05).

|  |  | CpG sites with mQTL | CpG sites without mQTL | Fold enrichment | p-value |
| --- | --- | --- | --- | --- | --- |
| IMAGE | Shelf | 7.13% | 7.44% | 0.9848 | 0.7278 |
|  | Shore | 11.97% | 12.15% | 0.9590 | 0.4411 |
|  | **CpG island** | **11.16%** | **14.33%** | **0.7788** | $\boldsymbol{1.056\times}\mathbf{10}^{\mathbf{-9}}$ |
|  | **Open sea** | **69.74%** | **66.08%** | **1.0554** | $\mathbf{0.0106}$ |
| IMAGE-I | Shelf | 7.51% | 7.44% | 1.0089 | 0.8923 |
|  | Shore | 11.58% | 12.17% | 0.9518 | 0.3848 |
|  | **CpG island** | **11.43%** | **14.17%** | **0.8067** | $\boldsymbol{7.415\times}\mathbf{10}^{\mathbf{-5}}$ |
|  | Open sea | 69.48% | 66.22% | 1.049 | 0.08541 |
| IMAGE-A | Shelf | 7.43% | 7.53% | 0.9877 | 0.8588 |
|  | Shore | 12.29% | 12.47% | 0.9862 | 0.7954 |
|  | **CpG island** | **12.94%** | **14.40%** | **0.8986** | **0.0202** |
|  | Open sea | 67.33% | 65.60% | 1.026 | 0.2981 |
| MACAU | Shelf | 7.37% | 7.39% | 0.9972 | 1.0000 |
|  | Shore | 12.04% | 12.21% | 0.9866 | 0.8381 |
|  | **CpG island** | **11.63%** | **14.20%** | **0.8190** | $\boldsymbol{6.542\times}\mathbf{10}^{\mathbf{-4}}$ |
|  | Open sea | 68.62% | 66.21% | 1.0364 | 0.2379 |
| GEMMA | Shelf | 7.52% | 7.41% | 1.0149 | 0.8455 |
|  | Shore | 11.95% | 12.21% | 0.9784 | 0.7559 |
|  | **CpG island** | **11.95%** | **14.15%** | **0.8445** | $\mathbf{0.0062}$ |
|  | Open sea | 68.59% | 66.23% | 1.0356 | 0.2804 |
| BB | Shelf | 7.50% | 7.40% | 1.0142 | 0.8322 |
|  | Shore | 11.58% | 12.20% | 0.9492 | 0.3810 |
|  | **CpG island** | **10.77%** | **14.13%** | **0.7622** | $\boldsymbol{2.434\times}\mathbf{10}^{\mathbf{-6}}$ |
|  | Open sea | 70.14% | 66.27% | 1.0584 | 0.0514 |

**Table S2**: Proportion of CpG sites directly disrupted by the SNP. The proportion of CpG sites that are directly disrupted by the SNP is higher for mQTL pairs versus non-mQTL pairs in the baboon data. Data are sorted in rows based on different association methods (IMAGE, IMAGE-I, IMAGE-A, MACAU, GEMMA, BB). Note that IMAGE-A was applied to analyze 38,250 SNP-CpG pairs while the other methods were applied to analyze all 49,196 SNP-CpG pairs. P-values are calculated using Fisher’s exact tests.

|  | CpG sites with mQTL | CpG sites without mQTL | P-values |
| --- | --- | --- | --- |
| IMAGE | 3.72% | 0.43% | $\text{<2.2×}\text{10}^{\text{-16}}$ |
| IMAGE-I | 4.25% | 0.63% | $\text{<2.2×}\text{10}^{\text{-16}}$ |
| IMAGE-A | 1.49% | 0.70% | $\text{3.107×}\text{10}^{\text{-7}}$ |
| MACAU | 5.66% | 0.62% | $\text{<2.2×}\text{10}^{\text{-16}}$ |
| GEMMA | 4.35% | 0.70% | $\text{<2.2×}\text{10}^{\text{-16}}$ |
| BB | 4.55% | 0.63% | $\text{<2.2×}\text{10}^{\text{-16}}$ |

**Table S3**: Enrichment of CpG sites in different genomic regions for CpG sites with mQTL versus CpG sites without mQTL in the wolf data. Percentage of CpG sites in different genomic regions (open sea; shelf; shore; CpG island) for CpG sites with mQTL (3^rd^ column) versus CpG sites without mQTL (4^th^ column). A fold enrichment is computed as the odds ratio (5^th^ column) and tested through a Fisher’s exact test (6^th^ column). mQTL are detected by different methods (IMAGE, IMAGE-I, IMAGE-A, MACAU, GEMMA, BB), based on a permutation-based empirical false discovery rate of 0.05. Bold font highlights significant enrichment (p-value < 0.05).

|  |  | CpG sites with mQTL | CpG sites with no mQTL | Fold enrichment | p-values |
| --- | --- | --- | --- | --- | --- |
| IMAGE | **Shelf** | **12.49%** | **11.62%** | **1.075** | $\boldsymbol{9.001\times}\mathbf{10}^{\mathbf{-5}}$ |
|  | **Shore** | **25.57%** | **24.64%** | **1.038** | $\boldsymbol{5.890\times}\mathbf{10}^{\mathbf{-3}}$ |
|  | **CpG island** | **30.17%** | **37.43%** | **0.8060** | $\boldsymbol{<2.2\times}\mathbf{10}^{\mathbf{-16}}$ |
|  | **Open sea** | **31.77%** | **26.31%** | **1.2075** | $\boldsymbol{<2.2\times}\mathbf{10}^{\mathbf{-16}}$ |
| IMAGE-I | **Shelf** | **13.42%** | **12.03%** | **1.1120** | $\boldsymbol{1.506\times}\mathbf{10}^{\mathbf{-3}}$ |
|  | Shore | 25.11% | 24.69% | 1.0173 | 0.5003 |
|  | **CpG island** | **32.42%** | **36.80%** | **0.8810** | $\boldsymbol{2.412\times}\mathbf{10}^{\mathbf{-8}}$ |
|  | **Open sea** | **29.05%** | **26.85%** | **1.0819** | $\boldsymbol{1.07\times}\mathbf{10}^{\mathbf{-3}}$ |
| IMAGE-A | Shelf | 11.62% | 11.82% | 0.9827 | 0.4198 |
|  | **Shore** | **24.08%** | **25.18%** | **0.9563** | $\boldsymbol{4.156\times}\mathbf{10}^{\mathbf{-3}}$ |
|  | CpG island | 35.34% | 35.88% | 0.9849 | 0.2589 |
|  | **Opea sea** | **28.96%** | **24.21%** | **1.1962** | $\boldsymbol{<2.2\times}\mathbf{10}^{\mathbf{-16}}$ |
| MACAU | **Shelf** | **13.49%** | **11.64%** | **1.1586** | $\boldsymbol{5.805\times}\mathbf{10}^{\mathbf{-6}}$ |
|  | Shore | 25.62% | 24.67% | 1.0383 | 0.124 |
|  | **CpG island** | **32.34%** | **36.66%** | **0.8822** | $\boldsymbol{1.295\times}\mathbf{10}^{\mathbf{-8}}$ |
|  | **Open sea** | **28.54%** | **27.03%** | **1.0559** | **0.0197** |
| GEMMA | **Shelf** | **13.48%** | **11.67%** | **1.1551** | $\boldsymbol{3.662\times}\mathbf{10}^{\mathbf{-4}}$ |
|  | Shore | 25.35% | 24.69% | 1.0267 | 0.391 |
|  | **CpG island** | **31.82%** | **36.61%** | **0.8962** | $\boldsymbol{3.437\times}\mathbf{10}^{\mathbf{-7}}$ |
|  | **Open sea** | **29.35%** | **27.03%** | **1.0858** | $\boldsymbol{4.393\times}\mathbf{10}^{\mathbf{-3}}$ |
| BB | Shelf | **12.98%** | **11.65%** | **1.1140** | $\boldsymbol{7.845\times}\mathbf{10}^{\mathbf{-4}}$ |
|  | Shore | 25.40% | 24.65% | 1.0302 | 0.214 |
|  | **CpG island** | **32.93%** | **36.65%** | **0.8985** | $\boldsymbol{6.143\times}\mathbf{10}^{\mathbf{-7}}$ |
|  | **Open sea** | **28.70%** | **27.05%** | **1.0610** | $\mathbf{0.0101}$ |

**Table S4**: Proportion of CpG sites directly disrupted by the SNP. The proportion of CpG sites that are directly disrupted by the SNP is higher in the associated SNP-CpG pairs versus the non-associated SNP-CpG pairs in the wolf data. Data are sorted in rows based on different association methods (IMAGE, IMAGE-I, IMAGE-A, MACAU, GEMMA, BB). Note that IMAGE-A was applied to analyze 236,092 SNP-CpG pairs while the other methods were applied to analyze all 279,223 SNP-CpG pairs. P-values are calculated through Fisher exact test.

|  | CpG sites with mQTL | CpG sites with no mQTL | P-values |
| --- | --- | --- | --- |
| IMAGE | 3.66% | 0.18% | $\text{<2.2×}\text{10}^{\text{-16}}$ |
| IMAGE-I | 8.06% | 0.34% | $\text{<2.2×}\text{10}^{\text{-16}}$ |
| IMAGE-A | 0.83% | 0.57% | $\text{2.14×}\text{10}^{\text{-6}}$ |
| MACAU | 8.91% | 0.33% | $\text{<2.2×}\text{10}^{\text{-16}}$ |
| GEMMA | 7.62% | 0.38% | $\text{<2.2×}\text{10}^{\text{-16}}$ |
| BB | 8.25% | 0.33% | $\text{<2.2×}\text{10}^{\text{-16}}$ |

**Table S5**: Type I error control of different methods in the null simulations across three different background heritability values. The type I error of different methods (IMAGE, IMAGE-I, IMAGE-A, MACAU, GEMMA, and BB) are shown at significance thresholds ranging from 0.05 to $1\times{10}^{-5}$.

|  | Method | $5\times{10}^{-2}$ | $1\times{10}^{-2}$ | $5\times{10}^{-3}$ | $1\times{10}^{-3}$ | $5\times{10}^{-4}$ | $1\times{10}^{-4}$ | $5\times{10}^{-5}$ | $1\times{10}^{-5}$ |
| --- | --- | --- | --- | --- | --- | --- | --- | --- | --- |
| $h^{2}=0$ | IMAGE | $5.16\times{10}^{-2}$ | 1.14$\times{10}^{-2}$ | 5.92$\times{10}^{-3}$ | 1.35$\times{10}^{-3}$ | 6.2$\times{10}^{-4}$ | 1.8$\times{10}^{-4}$ | 1.2$\times{10}^{-4}$ | 6$\times{10}^{-5}$ |
|  | IMAGE-I | 5.29$\times{10}^{-2}$ | 1.11$\times{10}^{-2}$ | 5.53$\times{10}^{-3}$ | 1.11$\times{10}^{-3}$ | 5.5$\times{10}^{-4}$ | 1.1$\times{10}^{-4}$ | 8$\times{10}^{-5}$ | 0 |
|  | IMAGE-A | 6.28$\times{10}^{-2}$ | 1.61$\times{10}^{-2}$ | 9.15$\times{10}^{-3}$ | 2.56$\times{10}^{-3}$ | 1.59$\times{10}^{-3}$ | 4.7$\times{10}^{-4}$ | 3$\times{10}^{-4}$ | 1.5$\times{10}^{-4}$ |
|  | MACAU | 4.74$\times{10}^{-2}$ | 9.28$\times{10}^{-3}$ | 4.49$\times{10}^{-3}$ | 8.7$\times{10}^{-4}$ | 4.3$\times{10}^{-4}$ | 1.1$\times{10}^{-4}$ | 5$\times{10}^{-5}$ | 0 |
|  | GEMMA | 4.88$\times{10}^{-2}$ | 9.41$\times{10}^{-3}$ | 4.55$\times{10}^{-3}$ | 7.4$\times{10}^{-4}$ | 3.4$\times{10}^{-4}$ | 7$\times{10}^{-5}$ | 5$\times{10}^{-5}$ | 0 |
|  | BB | 5.38$\times{10}^{-2}$ | 1.07$\times{10}^{-2}$ | 5.37$\times{10}^{-3}$ | 9.9$\times{10}^{-4}$ | 4.4$\times{10}^{-4}$ | 1$\times{10}^{-4}$ | 5$\times{10}^{-5}$ | 0 |
| $h^{2}=0.3$ | IMAGE | 5.38$\times{10}^{-2}$ | 1.16$\times{10}^{-2}$ | 6.44$\times{10}^{-3}$ | 1.62$\times{10}^{-3}$ | 9$\times{10}^{-4}$ | 1.9$\times{10}^{-4}$ | 8$\times{10}^{-5}$ | 0 |
|  | IMAGE-I | 5.45$\times{10}^{-2}$ | 1.21$\times{10}^{-2}$ | 6.86$\times{10}^{-3}$ | 1.67$\times{10}^{-3}$ | 1.05$\times{10}^{-3}$ | 3.5$\times{10}^{-4}$ | 1.6$\times{10}^{-4}$ | 4$\times{10}^{-5}$ |
|  | IMAGE-A | 6.23$\times{10}^{-2}$ | 1.54$\times{10}^{-2}$ | 8.64$\times{10}^{-3}$ | 2.55$\times{10}^{-3}$ | 1.5$\times{10}^{-3}$ | 6.6$\times{10}^{-4}$ | 4.1$\times{10}^{-4}$ | 1.2$\times{10}^{-4}$ |
|  | MACAU | 4.87$\times{10}^{-2}$ | 1.02$\times{10}^{-2}$ | 5.57$\times{10}^{-3}$ | 1.37$\times{10}^{-3}$ | 8.1$\times{10}^{-4}$ | 2.8$\times{10}^{-4}$ | 1.4$\times{10}^{-4}$ | 4$\times{10}^{-5}$ |
|  | GEMMA | 5.12$\times{10}^{-2}$ | 1.02$\times{10}^{-2}$ | 5.15$\times{10}^{-3}$ | 1.1$\times{10}^{-3}$ | 5.2$\times{10}^{-4}$ | 6$\times{10}^{-5}$ | 2$\times{10}^{-5}$ | 1$\times{10}^{-5}$ |
|  | BB | 5.72$\times{10}^{-2}$ | 1.27$\times{10}^{-2}$ | 6.77$\times{10}^{-3}$ | 1.52$\times{10}^{-3}$ | 9.1$\times{10}^{-4}$ | 2.5$\times{10}^{-4}$ | 1.6$\times{10}^{-4}$ | 3$\times{10}^{-5}$ |
| $h^{2}=0.6$ | IMAGE | 5.25$\times{10}^{-2}$ | 1.15$\times{10}^{-2}$ | 6.02$\times{10}^{-3}$ | 1.3$\times{10}^{-3}$ | 8.1$\times{10}^{-4}$ | 1.8$\times{10}^{-4}$ | 1.1$\times{10}^{-4}$ | 5$\times{10}^{-5}$ |
|  | IMAGE-I | 5.44$\times{10}^{-2}$ | 1.21$\times{10}^{-2}$ | 6.36$\times{10}^{-3}$ | 1.34$\times{10}^{-3}$ | 7.5$\times{10}^{-4}$ | 1.9$\times{10}^{-4}$ | 1$\times{10}^{-4}$ | 5$\times{10}^{-5}$ |
|  | IMAGE-A | 5.94$\times{10}^{-2}$ | 1.54$\times{10}^{-2}$ | 8.65$\times{10}^{-3}$ | 2.36$\times{10}^{-3}$ | 1.41$\times{10}^{-3}$ | 4.2$\times{10}^{-4}$ | 2.7$\times{10}^{-4}$ | 7$\times{10}^{-5}$ |
|  | MACAU | 4.83$\times{10}^{-2}$ | 1.02$\times{10}^{-2}$ | 5.04$\times{10}^{-3}$ | 1.03$\times{10}^{-3}$ | 5.9$\times{10}^{-4}$ | 1.1$\times{10}^{-4}$ | 8$\times{10}^{-5}$ | 4$\times{10}^{-5}$ |
|  | GEMMA | 5.17$\times{10}^{-2}$ | 9.9$\times{10}^{-3}$ | 4.97$\times{10}^{-3}$ | 9.7$\times{10}^{-4}$ | 5.1$\times{10}^{-4}$ | 9$\times{10}^{-5}$ | 4$\times{10}^{-5}$ | 1$\times{10}^{-5}$ |
|  | BB | 5.99$\times{10}^{-2}$ | 1.31$\times{10}^{-2}$ | 6.95$\times{10}^{-3}$ | 1.57$\times{10}^{-3}$ | 8.5$\times{10}^{-4}$ | 1.7$\times{10}^{-4}$ | 9$\times{10}^{-5}$ | 4$\times{10}^{-5}$ |

**Table S6**: Type I error control of different methods in the null simulations across three different sample sizes. The type I error of different methods (IMAGE, IMAGE-I, IMAGE-A, MACAU, GEMMA, and BB) are shown at significance thresholds ranging from 0.05 to $1\times{10}^{-5}$.

|  | Method | $5\times{10}^{-2}$ | $1\times{10}^{-2}$ | $5\times{10}^{-3}$ | $1\times{10}^{-3}$ | $5\times{10}^{-4}$ | $1\times{10}^{-4}$ | $5\times{10}^{-5}$ | $1\times{10}^{-5}$ |
| --- | --- | --- | --- | --- | --- | --- | --- | --- | --- |
| $n=50$ | IMAGE | 5.58$\times{10}^{-2}$ | 1.28$\times{10}^{-2}$ | 7.10$\times{10}^{-3}$ | 1.$78\times{10}^{-3}$ | 9$.7\times{10}^{-4}$ | 3.$0\times{10}^{-4}$ | $1.3\times{10}^{-4}$ | 6$\times{10}^{-5}$ |
|  | IMAGE-I | 5.89$\times{10}^{-2}$ | 1.46$\times{10}^{-2}$ | 8.50$\times{10}^{-3}$ | $2.83\times{10}^{-3}$ | 1.99$\times{10}^{-3}$ | 1.15$\times{10}^{-3}$ | 9.$7\times{10}^{-4}$ | $8.2\times{10}^{-4}$ |
|  | IMAGE-A | 7.05$\times{10}^{-2}$ | 2.07$\times{10}^{-2}$ | 1.27$\times{10}^{-2}$ | 4.32$\times{10}^{-3}$ | 2.62$\times{10}^{-3}$ | 9.4$\times{10}^{-4}$ | 6.0$\times{10}^{-4}$ | 1.8$\times{10}^{-4}$ |
|  | MACAU | 4.65$\times{10}^{-2}$ | 1.01$\times{10}^{-2}$ | 5.38$\times{10}^{-3}$ | 1.37$\times{10}^{-3}$ | 7.2$\times{10}^{-4}$ | 2.$0\times{10}^{-4}$ | 9$\times{10}^{-5}$ | 2$\times{10}^{-5}$ |
|  | GEMMA | 5.05$\times{10}^{-2}$ | 9.60$\times{10}^{-3}$ | 4.70$\times{10}^{-3}$ | 9.3$\times{10}^{-4}$ | 4.6$\times{10}^{-4}$ | 8$\times{10}^{-5}$ | $1\times{10}^{-5}$ | 0 |
|  | BB | 5.95$\times{10}^{-2}$ | 1.35$\times{10}^{-2}$ | 7.05$\times{10}^{-3}$ | 1.58$\times{10}^{-3}$ | 8.8$\times{10}^{-4}$ | 2.1$\times{10}^{-4}$ | 9$\times{10}^{-5}$ | 1$\times{10}^{-5}$ |
| $n=100$ | IMAGE | 5.38$\times{10}^{-2}$ | 1.16$\times{10}^{-2}$ | 6.44$\times{10}^{-3}$ | 1.62$\times{10}^{-3}$ | 9$\times{10}^{-4}$ | 1.9$\times{10}^{-4}$ | 8$\times{10}^{-5}$ | 0 |
|  | IMAGE-I | 5.45$\times{10}^{-2}$ | 1.21$\times{10}^{-2}$ | 6.86$\times{10}^{-3}$ | 1.67$\times{10}^{-3}$ | 1.05$\times{10}^{-3}$ | 3.5$\times{10}^{-4}$ | 1.6$\times{10}^{-4}$ | 4$\times{10}^{-5}$ |
|  | IMAGE-A | 6.23$\times{10}^{-2}$ | 1.54$\times{10}^{-2}$ | 8.64$\times{10}^{-3}$ | 2.55$\times{10}^{-3}$ | 1.5$\times{10}^{-3}$ | 6.6$\times{10}^{-4}$ | 4.1$\times{10}^{-4}$ | 1.2$\times{10}^{-4}$ |
|  | MACAU | 4.87$\times{10}^{-2}$ | 1.02$\times{10}^{-2}$ | 5.57$\times{10}^{-3}$ | 1.37$\times{10}^{-3}$ | 8.1$\times{10}^{-4}$ | 2.8$\times{10}^{-4}$ | 1.4$\times{10}^{-4}$ | 4$\times{10}^{-5}$ |
|  | GEMMA | 5.12$\times{10}^{-2}$ | 1.02$\times{10}^{-2}$ | 5.15$\times{10}^{-3}$ | 1.1$\times{10}^{-3}$ | 5.2$\times{10}^{-4}$ | 6$\times{10}^{-5}$ | 2$\times{10}^{-5}$ | 1$\times{10}^{-5}$ |
|  | BB | 5.72$\times{10}^{-2}$ | 1.27$\times{10}^{-2}$ | 6.77$\times{10}^{-3}$ | 1.52$\times{10}^{-3}$ | 9.1$\times{10}^{-4}$ | 2.5$\times{10}^{-4}$ | 1.6$\times{10}^{-4}$ | 3$\times{10}^{-5}$ |
| $n=150$ | IMAGE | $5.06\times{10}^{-2}$ | 1.07$\times{10}^{-2}$ | 5.06$\times{10}^{-3}$ | 1.14$\times{10}^{-3}$ | 6.1$\times{10}^{-4}$ | 1.9$\times{10}^{-4}$ | 8$\times{10}^{-5}$ | 4$\times{10}^{-5}$ |
|  | IMAGE-I | 5.20$\times{10}^{-2}$ | 1.32$\times{10}^{-2}$ | 6.03$\times{10}^{-3}$ | 1.39$\times{10}^{-3}$ | 7.5$\times{10}^{-4}$ | 2.1$\times{10}^{-4}$ | 1.4$\times{10}^{-4}$ | 4$\times{10}^{-5}$ |
|  | IMAGE-A | 5.66$\times{10}^{-2}$ | 1.61$\times{10}^{-2}$ | 7.25$\times{10}^{-3}$ | 1.75$\times{10}^{-3}$ | 9.4$\times{10}^{-4}$ | 3.0$\times{10}^{-4}$ | 2$\times{10}^{-4}$ | 5$\times{10}^{-5}$ |
|  | MACAU | 4.77$\times{10}^{-2}$ | 9.47$\times{10}^{-3}$ | 5.29$\times{10}^{-3}$ | 1.17$\times{10}^{-3}$ | 5.8$\times{10}^{-4}$ | 1.8$\times{10}^{-4}$ | 1$\times{10}^{-4}$ | 2$\times{10}^{-5}$ |
|  | GEMMA | 5.03$\times{10}^{-2}$ | 9.66$\times{10}^{-3}$ | 4.58$\times{10}^{-3}$ | 9.2$\times{10}^{-4}$ | 4.6$\times{10}^{-4}$ | 9$\times{10}^{-5}$ | 3$\times{10}^{-5}$ | 0 |
|  | BB | 5.54$\times{10}^{-2}$ | 1.21$\times{10}^{-2}$ | 6.59$\times{10}^{-3}$ | 1.55$\times{10}^{-3}$ | 7.6$\times{10}^{-4}$ | 1$.9\times{10}^{-4}$ | 1.2$\times{10}^{-4}$ | 3$\times{10}^{-5}$ |

**Table S7**: Type I error control of different methods in the null simulations across three different minor allele frequency. The type I error of different methods (IMAGE, IMAGE-I, IMAGE-A, MACAU, GEMMA, and BB) are shown at significance thresholds ranging from 0.05 to $1\times{10}^{-5}$.

|  | Method | $5\times{10}^{-2}$ | $1\times{10}^{-2}$ | $5\times{10}^{-3}$ | $1\times{10}^{-3}$ | $5\times{10}^{-4}$ | $1\times{10}^{-4}$ | $5\times{10}^{-5}$ | $1\times{10}^{-5}$ |
| --- | --- | --- | --- | --- | --- | --- | --- | --- | --- |
| $MAF=0.1$ | IMAGE | 5.44$\times{10}^{-2}$ | 1.28$\times{10}^{-2}$ | 7.16$\times{10}^{-3}$ | 1.95$\times{10}^{-3}$ | 1.16$\times{10}^{-3}$ | 3.3$\times{10}^{-4}$ | 2.4$\times{10}^{-4}$ | 5$\times{10}^{-5}$ |
|  | IMAGE-I | 5.31$\times{10}^{-2}$ | 1.15$\times{10}^{-2}$ | 6.04$\times{10}^{-3}$ | 1.44$\times{10}^{-3}$ | 7.9$\times{10}^{-4}$ | 2.$6\times{10}^{-4}$ | 1.6$\times{10}^{-4}$ | 4$\times{10}^{-5}$ |
|  | IMAGE-A | 7.47$\times{10}^{-2}$ | 2.24$\times{10}^{-2}$ | 1.43$\times{10}^{-2}$ | 5.19$\times{10}^{-3}$ | 3.29$\times{10}^{-3}$ | 1.2$\times{10}^{-3}$ | 8.5$\times{10}^{-4}$ | 4$\times{10}^{-4}$ |
|  | MACAU | 4.84$\times{10}^{-2}$ | 1$.03\times{10}^{-2}$ | 5.36$\times{10}^{-3}$ | 1.23$\times{10}^{-3}$ | 6.6$\times{10}^{-4}$ | 2$\times{10}^{-4}$ | 1.3$\times{10}^{-4}$ | 3$\times{10}^{-5}$ |
|  | GEMMA | 5.44$\times{10}^{-2}$ | 1.34$\times{10}^{-2}$ | 7.85$\times{10}^{-3}$ | 2.4$\times{10}^{-3}$ | 1.46$\times{10}^{-3}$ | 4$\times{10}^{-4}$ | 3$\times{10}^{-4}$ | 8$\times{10}^{-5}$ |
|  | BB | 5.45$\times{10}^{-2}$ | 1.14$\times{10}^{-2}$ | 5.86$\times{10}^{-3}$ | 1.29$\times{10}^{-3}$ | 6.8$\times{10}^{-4}$ | 2.2$\times{10}^{-4}$ | 9$\times{10}^{-5}$ | 3$\times{10}^{-5}$ |
| $MAF=0.3$ | IMAGE | 5.38$\times{10}^{-2}$ | 1.16$\times{10}^{-2}$ | 6.44$\times{10}^{-3}$ | 1.62$\times{10}^{-3}$ | 9$\times{10}^{-4}$ | 1.9$\times{10}^{-4}$ | 8$\times{10}^{-5}$ | 0 |
|  | IMAGE-I | 5.45$\times{10}^{-2}$ | 1.21$\times{10}^{-2}$ | 6.86$\times{10}^{-3}$ | 1.67$\times{10}^{-3}$ | 1.05$\times{10}^{-3}$ | 3.5$\times{10}^{-4}$ | 1.6$\times{10}^{-4}$ | 4$\times{10}^{-5}$ |
|  | IMAGE-A | 6.23$\times{10}^{-2}$ | 1.54$\times{10}^{-2}$ | 8.64$\times{10}^{-3}$ | 2.55$\times{10}^{-3}$ | 1.5$\times{10}^{-3}$ | 6.6$\times{10}^{-4}$ | 4.1$\times{10}^{-4}$ | 1.2$\times{10}^{-4}$ |
|  | MACAU | 4.87$\times{10}^{-2}$ | 1.02$\times{10}^{-2}$ | 5.57$\times{10}^{-3}$ | 1.37$\times{10}^{-3}$ | 8.1$\times{10}^{-4}$ | 2.8$\times{10}^{-4}$ | 1.4$\times{10}^{-4}$ | 4$\times{10}^{-5}$ |
|  | GEMMA | 5.12$\times{10}^{-2}$ | 1.02$\times{10}^{-2}$ | 5.15$\times{10}^{-3}$ | 1.1$\times{10}^{-3}$ | 5.2$\times{10}^{-4}$ | 6$\times{10}^{-5}$ | 2$\times{10}^{-5}$ | 1$\times{10}^{-5}$ |
|  | BB | 5.72$\times{10}^{-2}$ | 1.27$\times{10}^{-2}$ | 6.77$\times{10}^{-3}$ | 1.52$\times{10}^{-3}$ | 9.1$\times{10}^{-4}$ | 2.5$\times{10}^{-4}$ | 1.6$\times{10}^{-4}$ | 3$\times{10}^{-5}$ |
| $MAF=0.5$ | IMAGE | 5.05$\times{10}^{-2}$ | 1.08$\times{10}^{-2}$ | 5.7$\times{10}^{-3}$ | 1.1$\times{10}^{-3}$ | 6.4$\times{10}^{-4}$ | 1$.8\times{10}^{-4}$ | 1$\times{10}^{-4}$ | 3$\times{10}^{-5}$ |
|  | IMAGE-I | 5.65$\times{10}^{-2}$ | 1.25$\times{10}^{-2}$ | 6.45$\times{10}^{-3}$ | 1.46$\times{10}^{-3}$ | 8.5$\times{10}^{-4}$ | 1.9$\times{10}^{-4}$ | 1.2$\times{10}^{-4}$ | 5$\times{10}^{-5}$ |
|  | IMAGE-A | 5.82$\times{10}^{-2}$ | 1.34$\times{10}^{-2}$ | 7.6$\times{10}^{-3}$ | 1.91$\times{10}^{-3}$ | 9.4$\times{10}^{-4}$ | 2.8$\times{10}^{-4}$ | 1.9$\times{10}^{-4}$ | 5$\times{10}^{-5}$ |
|  | MACAU | 4.8$\times{10}^{-2}$ | 9.87$\times{10}^{-3}$ | 4.7$\times{10}^{-3}$ | 1.08$\times{10}^{-3}$ | 5.9$\times{10}^{-4}$ | 1.1$\times{10}^{-4}$ | 5$\times{10}^{-5}$ | 1$\times{10}^{-5}$ |
|  | GEMMA | 4.95$\times{10}^{-2}$ | 8.82$\times{10}^{-3}$ | 4.08$\times{10}^{-3}$ | 6.6$\times{10}^{-4}$ | 3.9$\times{10}^{-4}$ | 8$\times{10}^{-5}$ | 4$\times{10}^{-5}$ | 0 |
|  | BB | 6.23$\times{10}^{-2}$ | 1.42$\times{10}^{-2}$ | 7.33$\times{10}^{-3}$ | 1.74$\times{10}^{-3}$ | 9.3$\times{10}^{-4}$ | 1.8$\times{10}^{-4}$ | 1$\times{10}^{-4}$ | 4$\times{10}^{-5}$ |
