# Supplementary material for: IMAGE: high-powered detection of genetic effects on DNA methylation using integrated methylation QTL mapping and allele-specific analysis
Source: Genome Biol. 2019 Oct 24;20:220. doi: 10.1186/s13059-019-1813-1 (PMC6813132; doi:10.1186/s13059-019-1813-1)
Supplement: Supplementary file 4 — Additional file 4: Review history. [file 13059_2019_1813_MOESM4_ESM.docx]

Review History

**First round of review**

**Reviewer 1**

**Were you able to assess all statistics in the manuscript, including the appropriateness of statistical tests used? No**

**Were you able to directly test the methods? No**

**Comments to author:**

methods for methylation QTL mapping, IMAGE additionally incorporates allele-specific measures of DNA methylation which have the advantage of controlling for trans-acting genetic and environmental influences (as previously shown for measures of allele-specific expression). The manuscript is well-written and the method is likely to be very useful to the research community. I have only minor queries / comments:

1.     Because the total number of reads mapping to each allele are used to derive a proportion of methylated reads, the approach doesn't appear to suffer from the potential problem of read-mapping biases that can affect measures of allele-specific expression through RNA-Seq. I think this is a strength of the method, so the authors might wish to mention it.

2.     Using simulations, the authors demonstrate that the relative power of allele-specific methylation (vs mQTL) is greater when the narrow-sense heritability of the methylation status is increased. I understand that this will be due to confounding of the cis signal when there are greater trans-acting genetic influences on methylation. However, I find it counterintuitive that a trait that has no heritability (i.e. h2 = 0, as given in the example) can have any cis-genetic component to identify. This could be my misunderstanding, but I suggest explaining this more fully in the text for similarly confused readers.

3.     Some of the references appear to be missing page numbers.

**Reviewer 2**

**Were you able to assess all statistics in the manuscript, including the appropriateness of statistical tests used? Yes**

**Were you able to directly test the methods? Yes**

**Comments to author:**

In this paper, the authors describe a new method, IMAGE, for mapping methylation QTLs. This method is more advanced than existing approaches, and obtains a substantial boost in power by jointly modeling allele-specific methylation and total methylation using whole-genome (or reduce representation) bisulfite sequencing reads. The paper is well-written, and the method is state-of-the-art and substantially out-performs other approaches. The authors apply the method to datasets from a baboon population and a wolf population. I found a few things in the paper confusing and have some comments related to this.

1. Figure 2: Does the left-most panel correspond to the "baseline" simulated conditons in both (A) and (B) and if so, why are the results slightly different between panels (is this because the data from separate simulation runs)?

2. Figure 2: The panels would be more comparable if the y-axis were kept the same.

3. I am surprised that the IMAGE method is so much more powerful than the IMAGE-A method on the simulated data. I find this counterintuitive, because IMAGE is essentially a combination of IMAGE-I and IMAGE-A, but the power of these individual methods appears to be very low compared to IMAGE under most simulation conditions. This also seems inconsistent with the results presented in Fig. 3 and Fig. 4 (the baboon and wolf datasets), where the IMAGE-A method appears to have roughly 50% of the power of the combined IMAGE method. Is the explanation for the low power of IMAGE-A in Fig. 2 that the power analysis includes many sites that are not heterozygous in many individuals (because of low MAF)? Can this be explained in the text?

4. I found the description of h^2 as heritability to be confusing. I think this might be better described as "polygenic heritability", because the methylation of a CpG could be highly heritable (i.e. have high h^2) but be controlled by only a single SNP. In the single-SNP case, high-heritability would imply greater PVE and would improve the power of most approaches, not reduce power. I think what you really mean when you say h^2, is genetic background or polygenic heritability.

5. I was not familiar with the terms "Shelf", "Shore", "Open Sea". While I could look up their meaning, and they might be common terms in the methylation field, I would strongly suggest that these terms be defined more clearly in the main text or even included as a figure panel.

6. Related to (5) I did not know the meaning of "North Shelf" vs. "South Shelf" etc. If "North" and "South" in this context mean upstream or downstream of a CpG island, then I do not understand why they should be treated separately, since CpG islands (at least on their own) do not have an inherent directionality. This also leads to another question, which is why do the "S" and "N" enrichments appear to be in opposite directions from one another in Figure 3C (they are not in Fig 4C)? 

7. It seems like an important limitation of the IMAGE approach that, in unphased samples, only SNPs that fall within the same read (or read pair) can be tested for association with the methylation of a CpG. This limitation should be discussed further. I also think that it should be possible to work with more distant SNPs even in the case where the phasing is unknown (e.g. using a similar approach to EAGLE by Knowles et al. 2017.

8. It would be useful to define h^2 and \rho in the Figure 2 legend.

9. While I was able to install and run IMAGE, I had difficulties installing due to undocumented dependencies on other R packages including: foreach, doParallel, Matrix, MASS. These should be mentioned in the readme. I also received many warnings like this during the package installation: :Warning in rbinom(1, data[[4]][j, i], pi2[j]) : NAs produced"

**Authors Response**

**Point-by-point responses to the reviewers’ comments:**

Reviewer #1

This manuscript describes a statistical method (IMAGE) for mapping variants associated with altered DNA methylation using bisulfite sequencing data. In contrast to standard methods for methylation QTL mapping, IMAGE additionally incorporates allele-specific measures of DNA methylation which have the advantage of controlling for trans-acting genetic and environmental influences (as previously shown for measures of allele-specific expression). The manuscript is well-written and the method is likely to be very useful to the research community. I have only minor queries / comments: 

1. Because the total number of reads mapping to each allele are used to derive a proportion of methylated reads, the approach doesn't appear to suffer from the potential problem of read-mapping biases that can affect measures of allele-specific expression through RNA-Seq. I think this is a strength of the method, so the authors might wish to mention it. 

*Thank you for the comment. We fully agree. Following your suggestion, we have added a short note on this advantage in the Discussion section (lines 472-478 on page 13).* 

2. Using simulations, the authors demonstrate that the relative power of allele-specific methylation (vs mQTL) is greater when the narrow-sense heritability of the methylation status is increased. I understand that this will be due to confounding of the cis signal when there are greater trans-acting genetic influences on methylation. However, I find it counterintuitive that a trait that has no heritability (i.e. h2 = 0, as given in the example) can have any cis-genetic component to identify. This could be my misunderstanding, but I suggest explaining this more fully in the text for similarly confused readers. 

*Thanks, we apologize for the confusion. Our use of the term “heritability” for the h2 parameter was misleading: h2 actually represents the proportion of phenotypic variance explained by the genetic effects from all SNPs other than the SNP of focus (i.e the cis-SNP tested for association), and is more appropriately defined as “background heritability.” Thus, when h2 is zero, the focal SNP still has a cis-acting effect on DNA methylation (with effect size represented by the PVE), but all non-focal SNPs have effect sizes of zero. We have now clarified our definitions in the revised Materials and Methods section (lines 572-575 on page 17) and refer to h2 specifically as “background heritability.”* 

3. Some of the references appear to be missing page numbers. 

*Thank you for pointing out these errors, which we have fixed in the revised manuscript.*

Reviewer #2

In this paper, the authors describe a new method, IMAGE, for mapping methylation QTLs. This method is more advanced than existing approaches, and obtains a substantial boost in power by jointly modeling allele-specific methylation and total methylation using whole-genome (or reduce representation) bisulfite sequencing reads. The paper is well-written, and the method is state-of-the-art and substantially out-performs other approaches. The authors apply the method to datasets from a baboon population and a wolf population. I found a few things in the paper confusing and have some comments related to this. 

1. Figure 2: Does the left-most panel correspond to the "baseline" simulated conditions in both (A) and (B) and if so, why are the results slightly different between panels (is this because the data from separate simulation runs)? 

*Thank you for pointing out this potential source of confusion. In the revised Figure 2 legend, we now clarify that it is the middle panel in (A) (h2=0.3) and the left panel in (B) (rho=0) that correspond to the baseline simulated conditions (lines 1238-1239 on page 36). We have also added similar information to the figure legend for Supplementary Figure S1.* 

2. Figure 2: The panels would be more comparable if the y-axis were kept the same. 

*Thank you for the suggestion. We have updated Figure 2 to keep the y-axis the same in (A) and (B). We have made a parallel revision to Supplementary Figure S1.* 

3. I am surprised that the IMAGE method is so much more powerful than the IMAGE-A method on the simulated data. I find this counterintuitive, because IMAGE is essentially a combination of IMAGE-I and IMAGE-A, but the power of these individual methods appears to be very low compared to IMAGE under most simulation conditions. This also seems inconsistent with the results presented in Fig. 3 and Fig. 4 (the baboon and wolf datasets), where the IMAGE-A method appears to have roughly 50% of the power of the combined IMAGE method. Is the explanation for the low power of IMAGE-A in Fig. 2 that the power analysis includes many sites that are not heterozygous in many individuals (because of low MAF)? Can this be explained in the text? 

*Thank you for the comment; we did not explain this issue clearly in the previous version of the manuscript. The higher power of IMAGE vs IMAGE-A seen in the simulations as compared to in the real data applications is mostly due to the choice of two parameters in the simulated data: PVE and h2. Specifically, in the baseline simulation setting, we set PVE=0.10 and h2=0.3 (note that we also explored h2=0 or 0.6 and PVE=0.05 or 0.15). We chose h2=0.3 in the baseline simulation because h2=0.3 is close to the mean heritability estimate across all methylation sites in the baboon data (m=433,871). However, the methylation sites we examined in the real data are a subset of sites that have a SNP nearby (m=45,210) and these sites tend to have a high heritability estimate (median = 0.529 in the baboon data and 0.520 in the wolf data). Higher h2 tends to narrow the performance gap between IMAGE and IMAGE-A (e.g. third subpanel in Figure 2A, where h2=0.6). Similarly, we chose PVE=0.10 in the baseline simulation because we thought it would be a reasonable PVE across all associated SNP-CpG pairs. But because SNP-CpG pairs tested in the real data are a subset of all pairs—specifically, those that tend to have SNPs closer to the focal CpG site than average—the estimated PVE is higher in the real data applications (median PVE estimate for the subset of sites tested is 0.152 in the baboon data and 0.159 in the wolf data). A higher PVE can also lead to a narrower performance gap between IMAGE and IMAGE-A (e.g. third subpanel in Supplementary Figure S1B). 

We have added a short paragraph in the Results section to explain this important issue (lines 307-315 on page 9). We also now empirically demonstrate that the differences in h2 and PVE between the real and simulated data completely explain the difference in relative performance between IMAGE and IMAGE-A across settings. To do so, we created a new simulation setting using h2=0.5 and PVE=0.15. The new simulation results replicate the power difference among different methods in the real data. For example, the power of IMAGE-A is approximately 60.7% of IMAGE, close to what we see in the wolf and baboon data sets. The new results are referenced in lines 314-315 and shown in Supplementary Figure S1G.* 

4. I found the description of h^2 as heritability to be confusing. I think this might be better described as "polygenic heritability", because the methylation of a CpG could be highly heritable (i.e. have high h^2) but be controlled by only a single SNP. In the single-SNP case, high-heritability would imply greater PVE and would improve the power of most approaches, not reduce power. I think what you really mean when you say h^2, is genetic background or polygenic heritability. 

*This comment dovetails with Reviewer 1’s above, and we fully agree. In response to both comments, we have changed the term h2 from “heritability” to “background heritability” throughout the text. We felt that “background heritability” was slightly more precise than the reviewer’s other suggestion of “polygenic heritability”. In addition, we have provided a detailed definition and explanation of h2 in the revised Materials and Methods section (lines 572-575 on page 17).* 


5. I was not familiar with the terms "Shelf", "Shore", "Open Sea". While I could look up their meaning, and they might be common terms in the methylation field, I would strongly suggest that these terms be defined more clearly in the main text or even included as a figure panel. 

*Thank you for the comment. Following the suggestion, we have added a new illustration to Figure 3C and Figure 4C panel to show how these terms were defined. Briefly, CpG islands are defined as regions of high CpG density in an otherwise CpG-sparse genome [1]. CpG shores are defined as the 2 kb of sequence flanking a CpG island, with the upstream 2kb sequence denoted as the north shore and downstream 2kb sequence denoted as the south shore. CpG shelves are defined as the 2 kb of sequence further flanking CpG shores, with the 2kb sequence upstream of the north shore denoted as north shelf and 2kb sequence downstream of the south shore denoted as south shelf. Both CpG shores and shelves have been reported to be more dynamic than the CpG island itself [2-4], and have been associated with various phenotypes and environmental perturbations. Finally, the remaining regions outside of CpG islands, shores, and shelves are denoted as open sea [5]. These definitions are now also provided in the updated Materials and Methods section (lines 815-824 on page 22).* 

6. Related to (5) I did not know the meaning of "North Shelf" vs. "South Shelf" etc. If "North" and "South" in this context mean upstream or downstream of a CpG island, then I do not understand why they should be treated separately, since CpG islands (at least on their own) do not have an inherent directionality. This also leads to another question, which is why do the "S" and "N" enrichments appear to be in opposite directions from one another in Figure 3C (they are not in Fig 4C)? 

*Thank you for the comment. Indeed, “North” and “South” represents upstream and downstream of a CpG island, respectively (see above). We initially followed previous studies and stratified north and south shores/shelves [6, 7], but agree with the reviewer that it does not make sense in the context of the current analysis to distinguish them. We have now merged the results for north and south regions together in the updated manuscript (lines 824-828 on page 23). We suspect that the non-significant but opposite trend of “N” versus “S” enrichments in the earlier version of our manuscript was due to the small number of CpG sites (m=45,210) in the baboon data and a corresponding lack of statistical power. With the much larger number of CpG sites in the wolf data (m=242,784), the “S” and “N” enrichments were both directionally consistent and statistically significant (original Figure 4C). Although we have combined “N” and “S” shores and shelves in the revision, we also now point out the difference in CpG site numbers as a likely source of power differences for enrichment analysis between the two data sets (lines 425-427 on page 12).* 

7. It seems like an important limitation of the IMAGE approach that, in unphased samples, only SNPs that fall within the same read (or read pair) can be tested for association with the methylation of a CpG. This limitation should be discussed further. I also think that it should be possible to work with more distant SNPs even in the case where the phasing is unknown (e.g. using a similar approach to EAGLE by Knowles et al. 2017. 

*Thank you for the comments. We fully agree. We have added a discussion on this important limitation together with a citation to [8] in the Discussion section (line 493-498 on page 14).* 


8. It would be useful to define h^2 and \rho in the Figure 2 legend. 

*Thank you. We have now defined these two parameters in the Figure 2 legend.* 


9. While I was able to install and run IMAGE, I had difficulties installing due to undocumented dependencies on other R packages including: foreach, doParallel, Matrix, MASS. These should be mentioned in the readme. I also received many warnings like this during the package installation: :Warning in rbinom(1, data[[4]][j, i], pi2[j]) : NAs produced" 

*Thank you. We have fixed these bugs in our software. We have also submitted the software to R CRAN to further enhance the usability of the software.* 

References 
1. Gardinergarden, M. and M. Frommer, Cpg Islands in Vertebrate Genomes. Journal of Molecular Biology, 1987. 196(2): p. 261-282. 
2. Doi, A., et al., Differential methylation of tissue- and cancer-specific CpG island shores distinguishes human induced pluripotent stem cells, embryonic stem cells and fibroblasts. Nature Genetics, 2009. 41(12): p. 1350-1353. 
3. Irizarry, R.A., et al., The human colon cancer methylome shows similar hypo- and hypermethylation at conserved tissue-specific CpG island shores. Nature Genetics, 2009. 41(2): p. 178-186. 
4. Bibikova, M., et al., High density DNA methylation array with single CpG site resolution. Genomics, 2011. 98(4): p. 288-295. 
5. Sandoval, J., et al., Validation of a DNA methylation microarray for 450,000 CpG sites in the human genome. Epigenetics, 2011. 6(6): p. 692-702. 
6. Olsson, A.H., et al., Genome-Wide Associations between Genetic and Epigenetic Variation Influence mRNA Expression and Insulin Secretion in Human Pancreatic Islets (vol 10, e1004735, 2014). Plos Genetics, 2014. 10(12). 
7. Volkov, P., et al., A Genome-Wide mQTL Analysis in Human Adipose Tissue Identifies Genetic Variants Associated with DNA Methylation, Gene Expression and Metabolic Traits. Plos One, 2016. 11(6): p. e0157776. 
8. Knowles, D.A., et al., Allele-specific expression reveals interactions between genetic variation and environment. Nature Methods, 2017. 14(7): p. 699-702.

**Second round of review**

**Reviewer 2**

The authors have addressed all of my concerns.
